# The Impact of Our Personality on Others: The Lithuanian Comprehensive Lexical Taxonomy of Social Effects

**DOI:** 10.3389/fpsyg.2022.869920

**Published:** 2022-04-25

**Authors:** Ana Volungevičienė, Boris Mlačić, Oleg Gorbaniuk

**Affiliations:** ^1^Department of General Psychology, Institute of Psychology, The John Paul II Catholic University of Lublin, Lublin, Poland; ^2^Institute of Social Sciences Ivo Pilar, Zagreb, Croatia

**Keywords:** social effects, psycholexical approach, Lithuanian language, self-ratings, observer-ratings

## Abstract

Social effects represent the psychological (emotional, cognitive, and motivational) reactions evoked in other people by the expression of traits in behavior and emotion. From the transactional view on personality, studying the psycholexical structures of social effects can help to discover unique vs. common thought and behavior patterns, affects, and motivations, which are primarily related to personality dispositions. Thus, we developed the comprehensive taxonomy of social effects following the principles of the psycholexical approach. In the first study, two judges selected 9,625 person-descriptive terms—adjectives, type-nouns, attribute-nouns, and participles—from the Dictionary of the Standard Lithuanian Language. In the second study, six judges classified all the selected descriptors using German psycholexical methodology. Finally, a principal component analysis was performed, followed by varimax rotation for the 208 social-effect descriptors, separately for ipsatized self-ratings and observer-ratings from 203 to 204 Lithuanian students, respectively. We found out that the five-component solution was the best fit for self-ratings, whereas for observer-ratings it was a four-component structure. In this article, we present the results from the factor analyses and discuss our findings in the context of previous studies, as well as cross-language personality models.

## Introduction

The psycholexical approach to personality is based on the lexical hypothesis (Goldberg, 1981) which assumes that the most important individual differences are encoded in the natural language. By constructing comprehensive lists of person characteristics in different languages, and factor analyzing self and/or observer-ratings on these lists, we can identify the fundamental cross-cultural and culturally specific dimensions of individual differences by which a person can be described.

In the lexical tradition, one of the most important aims was to structure the domain of person characteristics. Although the early taxonomies identified some categories beyond stable traits (refer to Allport and Odbert, 1936; Norman, 1967), the most comprehensive framework for naming, differentiating, and classifying individual differences was proposed by German scholars (Angleitner et al., 1990). These authors distinguished five superordinate categories that were broken down into thirteen subordinate classes describing temperamental and character attributes, abilities and talents, experiential, physical, and bodily states, observable activities, roles and relationships, social effects, pure evaluations, attitudes and worldviews, anatomy and constitution, appearance, context-specific or technical descriptors, and metaphorical terms. Since most lexical studies focused on examining the structure of dispositional attributes (e.g., Ostendorf, 1990; Caprara and Perugini, 1994; Szirmák and De Raad, 1994; Szarota, 1996; Hahn et al., 1999; Mlačić and Ostendorf, 2005; Hrebíčková, 2007; Gorbaniuk et al., 2013; Mai, 2014; Farahani et al., 2016; Livaniene and De Raad, 2016), the untapped potential of other personality-relevant categories was not fully exploited. Various characteristics apart from dispositional attributes could impact the way people think about themselves or others, and how people differ from each other (refer to De Raad and Mlačić, 2017).

The vast majority of lexical studies conducted, thus, far have defined personality in a relatively narrow way by focusing on the time-stable qualities containing psychological aspects. The resulting psycholexical structures of the natural languages primarily spoken in Europe to a greater or lesser extent appeared to confirm the cross-language replicability of the Big Five (Brokken, 1978; Goldberg, 1990; Ostendorf, 1990; De Raad, 1992; Szarota, 1996; Mlačić and Ostendorf, 2005; Hrebíčková, 2007) and the six-factor structure (Ashton et al., 2004). Later studies that focused on finding cross-language lexical dimensions provided evidence for a smaller number of recurring factors, namely the Big Three (De Raad et al., 2010, 2014) and the Big Two (Saucier et al., 2014; De Raad et al., 2018a).

Another group of lexical studies used a broader variable selection and went beyond dispositional attributes by also including such human qualities as the social and reputational aspects of personality, temporary conditions, or overt characteristics and appearance (Almagor et al., 1995; Benet and Waller, 1995; Saucier, 1997; Church et al., 1998; De Raad and Barelds, 2008; De Raad et al., 2018b; as cited in Saucier, 1997; Zhou et al., 2009). This strategy resulted in more elaborate six-factor, seven-factor, or eight-factor lexical structures. However, some of the studies mentioned above not only used a broader variable selection but also followed a different methodology in which scholars inspected either every fourth (Almagor et al., 1995; Benet and Waller, 1995) or tenth (De Raad et al., 2018b) page of a dictionary for relevant descriptors. As noted by De Raad and Mlačić (2017), this specific sampling might have resulted in the systematic exclusion of terms starting with prefixes, e.g., dis-, im-, in-, mis- or un-, and those referring to the lack of a certain personality characteristic. Thus, a relatively lower number of negative descriptors could potentially have affected the size of the negative poles of some of the uncovered dimensions. Overall, a broader variable selection, as well as methodological solutions, found additional personality dimensions, for example, Positive Valence and Negative Valence (Almagor et al., 1995; Benet-Martínez and Waller, 1997; Ademi Shala et al., 2020), Virtue, Competence, or Hedonism (De Raad and Barelds, 2008).

Although the majority of psycholexical studies focused on either narrowly or widely defined personality dispositions, some researchers examined other personality-relevant categories. Ostendorf (1996) was the first to factor analyze the German terms that referred to attitudes and worldviews and distinguished a two-factor structure encompassing Religiousness and Conservativism vs. Radicalism, both dimensions unrelated to the Big Five. Another study (Benet-Martínez and Waller, 2002) examined the structure of Spanish terms that described pure evaluations and detected five factors: Depravity, Distinction, Worthlessness, Unconventionality, and Stupidity. Whereas, Filipino scholars (Imperio et al., 2008) analyzed terms referring to social roles, statuses, social effects, as well as physical attributes, and identified ten factors, namely Prominence, Uselessness, Attractiveness, Respectability, Uniqueness, Destructiveness, Presentableness, Strength, Dangerousness, and Charisma. These authors concluded that social and physical attributes contained information relevant to personality, and vice-versa.

To date, the category of social effects was analyzed in the English and Croatian languages. The first investigation was performed by Saucier (2010) who collected 201 other-ratings and 700 self-ratings on the list of 32 prototypical social-effect descriptors in the English language. Raw and ipsatized data provided by both samples were factors analyzed separately, and the author opted for the two-factor structure. The first dimension of the English structure of social effects referred to being a source of pleasure to others, and the second factor described a person as a source of pain to others. The author concluded that the English structure of social effects corresponded to the Big Two personality structure.

The other study examined the social and reputational aspects of personality in the Croatian language, which, among other psycholexical subcategories, included social-effect descriptors (Mlačić, 2016). Ratings on the 138 social-effect adjectives were provided by 524 self-raters and 502 other-raters. Although ipsatized data were analyzed separately for the two perspectives, the optimal three-factor structure in both samples consisted of quite similar dimensions labeled as Attractiveness-Popularity, Mysteriousness-Irritation, and Likeability. Based on the content analysis, Mlačić (2016) concluded that the first two Croatian factors somewhat resembled the English factors of social effects. Correlations between the Croatian dimensions and the Big Five factors provided evidence for the pronounced relations between Attractiveness-Popularity and the Big Five Extraversion, Likeability and the Big Five Agreeableness, as well as Mysteriousness-Irritation and the Big Five Conscientiousness.

Although previous studies provided some evidence for the relationship between personality dispositions and social effects, the question about the capacity of the latter class to describe personality still remains. Early researchers (Allport and Odbert, 1936) suggested that terms denoting social evaluations and effects did not designate traits and should be avoided by psychologists because of their evaluative nature. However, if someone defines personality as the social influence of a person, then social-effect descriptors become central as they describe the social stimulus value of an individual. It should be noted that Allport and Odbert (1936) combined social effects and pure evaluations into one category, which is not fully justified. We can indeed argue that all the terms denoting individual differences carry an evaluative component. However, in the case of pure evaluations, the evaluative content dominates the descriptive component, and it is hard to use pure evaluations for descriptive purposes (refer to Norman, 1967). Whereas, social effects encode psychological reactions to personality dispositions, which means social-effect descriptors have a strong descriptive component providing information about the social stimulus value of an observed person. Although John (1990) differentiated between social effects and pure evaluations, he considered social effects to be effects evoked in others by expressing a particular trait in emotion or behavior and suggested that this class of descriptors was secondary to personality traits. Thus, social-effect descriptors obtained a status of avoidable and insignificant human qualities. An alternative approach was proposed by Saucier (2010) who assumed that personality dispositions could be constituted in both the observer and the perceiver. From this transactional point of view, personality dispositions are derived from the interaction between a person and their environment, especially the social environment. The social effect of an observed person on an observer may reflect a mixture of the behavioral patterns of the former and the motivational, emotional, and cognitive sets of the latter. Thus, social-effect terms could be more central to personality than previously expected.

Based on definitions from the psycholexical approach, personality dispositions, and social effects are somewhat related, however, it is crucial to differentiate between these two types of individual differences. Thus, personality dispositions are relatively time-stable and cross-situationally consistent qualities containing psychological aspects, whereas social effects are temporary psychological reactions to the expression of dispositions, which means social effects are states by their nature. Additionally, personality dispositions are constituted in the observed person, whereas social reactions are effects experienced by observers. At first sight, social reactions do not provide any information about the personality dispositions of the observed person, they just describe the effects that an unknown quality or set of such qualities have on the observer. For example, by stating that a person is “boring” we do not provide any information on what personal quality or qualities make us feel bored, however, we are certain that the observed individual does not make us feel entertained or excited. While remaining within the framework of “classically” described personality dispositions, we can still use categories of social effects to increment our understanding and assessment of personality. Therefore, by studying the social-effect structure on large samples and analyzing relations between social-effect and dispositional dimensions, we could discover how personality dispositions expressed by recurrent behavioral patterns interact with frequently occurring emotional, motivational, and cognitive reactions. If some recurrent relationships and patterns could be detected, this might suggest that our understanding of personality could shift from attributes “within” people to attributes “between” people. Also, personality assessment would have to include not only measurements of intrinsic attributes but also of emotional, motivational, and cognitive patterns closely related to personality dispositions.

As noted by Saucier (2010), the primary and superior source for social-effect data should be ratings provided by others. As observers, we know how other people affect us, and what emotional, motivational, and cognitive reactions they evoke in us. Thus, when providing other-ratings, respondents describe the states that they know relatively well. On the other hand, the self-rating perspective for social effects seems to be more challenging as participants provide their opinions on states experienced by other people, and not themselves. This involves memory processes and requires observations to be very careful and accurate. Also, the participant has to resist responding in a socially desirable manner and must average the reaction of many people who react to them. However, what is said above does not exclude self-ratings from the study, rather it emphasizes the superiority of other-ratings over self-ratings in the analyses of social-effect descriptors.

To exploit the full potential of the psycholexical approach, scholars should go beyond dispositional adjectives, which means including other personality-relevant categories (refer to De Raad and Mlačić, 2017), and using various word classes capable of describing personality (De Raad, 2000). Although adjectives are considered as having the greatest personality-descriptive capacity, especially for European languages (refer to Saucier, 2003), many studies showed that nouns (e.g., De Raad and Hoskens, 1990; Henss, 1998; Saucier, 2003; Di Blas, 2005) and verbs (De Raad et al., 1988; De Raad, 1992; Hrebíčková et al., 1999) uncover unique lexical factors beyond the adjective-based dimensions. Also, the research by De Raad and Barelds (2008) that included words of various classes (adjectives, adverbs, attribute-nouns, type-nouns, verbs, and short expressions) showed that although parts of speech other than adjectives did not constitute separate lexical factors, they enriched the content of the adjective-based dimensions by filling their segments with specific meaning (Barelds and De Raad, 2015). Thus, there is a need to use a comprehensive approach in terms of various word classes to uncover new aspects of individual differences.

The present research aims to explore the psycholexical structure of social-effect descriptors in the Lithuanian language. We define social effects as psychological (cognitive, emotional, or motivational) reactions to the expression of the personality dispositions of an observed person. In our study, the German method (Angleitner et al., 1990) and an approach similar to De Raad and Barelds (2008) were used to construct a comprehensive list of social-effect descriptors in terms of different word classes—adjectives, type-nouns, attribute-nouns, and participles. We examined indigenous self-rating and observer-rating structures at different levels of hierarchy and discussed our results in the context of previous psycholexical studies focused on the lexical structure of social-effect descriptors (Saucier, 2010; Mlačić, 2016). Also, we analyzed relations between indigenous structures and cross-language personality structures, namely the Big Five (Goldberg, 1990, 1992) and the HEXACO model originating from the lexical six-factor solution (Ashton and Lee, 2009).

## Materials and Methods

### Study 1: Construction of a Comprehensive List of Personality-Relevant Descriptors in the Lithuanian Language

#### Selection and Classification of Personality-Relevant Descriptors

Following the German approach (Angleitner et al., 1990), we enrolled eight judges (one of the authors and seven students in the final year of psychological studies) to independently scan the latest and currently most complete Dictionary of the Standard Lithuanian Language containing over 76,000 entries (Lithuanian Language Institute, 2012–2017). As in the German study (Angleitner et al., 1990), we divided the dictionary into seven parts to make the selection task less daunting for the assessors. Overall, the dictionary was analyzed independently by two judges: one of the authors scanned the entire dictionary, whereas the seven students worked on their assigned fragments. All the judges were instructed to extract personality-relevant adjectives, type-nouns, attribute-nouns, and participles that are used to describe human characteristics and make it possible to differentiate between people. At this stage, all the questionable terms were included on the list. The entire selection procedure, including inclusion and exclusion criteria, test questions for different word classes, as well as preparatory training, has been described in detail in a separate article (Ivanova et al., 2018).

Second, a total of six judges (one of the authors and five students in the final year of psychological studies) independently classified the 9,625 terms, which had been selected in the first step, into six superordinate categories: (1) dispositions; (2) temporary conditions; (3) social and reputational aspects; (4) overt characteristics and appearance; (5) specific terms; and (6) metaphors. We slightly modified a category of specific terms from the German classification system (Angleitner et al., 1990) by grouping the subclass of metaphorical terms into a separate category; otherwise, the class of specific terms could be too heterogeneous. The six superordinate categories comprised eleven subordinate categories. To assign a descriptor to a particular superordinate or subordinate category, at least four of the six judges had to classify it the same way. As in numerous psycholexical studies, we checked the inter-judge agreements of the classifications. According to the German classification system (Angleitner et al., 1990), the social-effect descriptors together with terms denoting roles and relationships, pure evaluations, and attitudes and worldviews, fall into the superordinate category of social and reputational aspects. Although the mean alpha coefficient for the superordinate category of social and reputational aspects was α = 0.92, the inter-judge consistency for social-effect descriptors reached a level of α = 0.56. The full report on the validity and consistency of classification decisions, as well as taxonomy results, was comprehensively presented in a separate article (Ivanova et al., 2018).

#### Refining the List of Social-Effect Descriptors

Completion of the classification task resulted in the initial pool of 164 social-effect terms. Since the inter-judge consistency for social-effect descriptors was relatively low, we took additional steps to ensure that the list of social effects was not missing important descriptors. Thus, two of the current study's authors checked the terms assigned to other categories of individual differences which could have been classified as such by mistake. As previous experience shows, social effects can be difficult to distinguish from other descriptors of human qualities. For example, Allport and Odbert (1936) classified social effects and social evaluations under the same category labeled Column III. Also, Saucier (2010) reported that some terms classified by Norman (1967) as social effects, were assigned in Saucier's study to the subcategory of social evaluations, appearance, or dispositions. Hence, after a thorough examination of the Lithuanian descriptors of individual differences, 113 terms were added to the list of social effects.

Additionally, one of the authors scanned the Dictionary of the Standard Lithuanian Language (Lithuanian Language Institute, 2012–2017) and selected 163 verbs denoting social effects. The main criteria for the selection were to include all of the verbs that denote either emotional, motivational, or cognitive reaction to the expression of personality disposition(s). Also, when making selection decisions, the author used several questions that were meant to facilitate the construction of the list (Gorbaniuk et al., 2019): (1) John is a person who often/rarely/never [verb] (e.g., to disappoint), (2) John is a person who can [verb] better/worse than Paul (e.g., to persuade), and (3) John often/rarely/never [verb] other people (e.g., to encourage). Another author of the study checked the resulting list for relevancy. It is worth noting that previous lexical studies on social-effect descriptors did not include verbs. This step resulted in a set of 440 social-effect descriptors.

According to some psycholexical researchers (Almagor et al., 1995; De Raad, 2000), the psycholexical approach is substandard when it does not include all the word classes capable of describing human qualities. In the present study, we aimed to construct a comprehensive list of social effects by retaining terms from different word classes that potentially denote human qualities. For this purpose, we used an approach similar to De Raad and Barelds (2008). After pooling all the relevant terms, it was important to reduce the morphemic redundancy understood as the word root and meaning repetition in various parts of speech. For example, among the terms *nuobodus* (boring, adjective), *nuoboda* (boring person, type-noun), *nuobodumas* (boredom, attribute-noun), and *nuobodŽiauti* (to be bored, verb), we have chosen an adjective as the best representative of social effects. We did not give priority to any of the word classes. Thus, we excluded 183 morphemically redundant descriptors and arrived at a pool of 257 terms. Then, we used the Corpus of the Contemporary Lithuanian Language (Centre of Computational Linguistics, Vytautas Magnus University, 1998–2013) to check the frequency of use of these 257 terms and removed 10 descriptors from the pool as their frequency of use was zero. Finally, we asked four native speakers to provide familiarity ratings on a Yes/No scale and excluded 23 descriptors unfamiliar to at least three of the four judges.

At this stage, the list consisted of 224 social-effect descriptors: 70 adjectives, 16 attribute-nouns, 1 type-noun, 136 verbs, and 1 short expression. Although at the descriptor selection stage we were not able to include participles in our list as this part of speech was not listed in the form of separate entries in the Dictionary of the Standard Lithuanian (Lithuanian Language Institute, 2012–2017), when refining the final list, we changed 122 verbs to participles—a word class that derives from verbs and carries features of both verbs and adjectives (refer to Quirk et al., 1972). This helped to emphasize the social effects denoted by some terms and better incorporate verbs in the list. Thus, the descriptor “to allure” (*vilioti*) was replaced by the word “alluring” (*viliojantis*). To include the remaining verbs in the list, we formulated sentences by adding a filler “I can” (*sugebu*) at the beginning. For example, I can [influence, amuse, persuade] [*Sugebu* (*paveikti, i̧linksminti, i̧kalbėti*)]. Also, we added appropriate fillers to incorporate attribute-nouns in the list. For instance, causing [tension] [*keliantis* (*i̧tampa̧*)] or giving [stimuli] [*suteikiantis* (*stimula̧*)].

Two of the current study's authors performed a classification of the terms and found that 47.78% of the social-effect descriptors denoted emotional reactions (e.g., exciting, *jaudinantis*), 25.45%—of cognitive reactions (e.g., confusing, *klaidinantis*), 16.96%—motivational reactions (e.g., irresistible, *pavergiantis*), and 12.95%—reputational aspects of social effects (e.g., appreciated, *brangus kitiems*), whereas 7.14% of terms were hard to classify in any category of the social-effect descriptors mentioned above (e.g., inaccessible, *neprieinamas*).^1^

To ensure cross-cultural comparisons, we compared the Lithuanian list of social-effect descriptors to markers for the English and Croatian social-effect factors. Most marker terms from previous studies were detected on the Lithuanian list, however, to attain a higher degree of internal consistency of the English and Croatian social-effect scales, we added 2 and 6 missing adjectives from the English and Croatian scales, respectively. Thus, the final list included 232 social-effect terms.

### Study 2: Factor Structure of Lithuanian Social-Effect Descriptors

In this study, we used five measures: (1) the list of Lithuanian social effects, (2) the Big Five measure, and (3) the HEXACO model measure. Markers for the (4) English (Saucier, 2010) and (5) Croatian (Mlačić, 2016) structures of social effects were included in the Lithuanian list of social effects.

#### Measures

##### The Lithuanian List of Social Effects

All the participants completed an inventory containing 232 descriptors of social effects. The terms were put in random order. The students were asked to use a 7-point scale (1 = very inaccurate to 7 = very accurate), or to respond with “0” when the meaning of a term was not fully clear to them. In the observer-rating sample, participants were instructed to describe either a man or woman they had known well for at least 2 years. In this study, we controlled three variables: (a) the attitude toward the target (negative, neutral, or positive) × (b) the gender of the participant (men or women) × (3) the gender of the target (men or women). We used quota sampling and allocated participants to random groups in terms of different instructions to control the attitude toward the target person. The negative attitude meant that participants had to describe a man (woman) of their age whom they had known well for at least 2 years and rather disliked. The students enrolled in the so-called neutral attitude group were instructed to describe a man (woman) of their age whom they knew well and toward whom they had a neutral attitude. Finally, the participants allocated to the positive attitude group were asked to describe a man (woman) of their age whom they had known well for at least 2 years and rather liked. Controlling the attitude toward the target person helped to collect descriptions that potentially reflected the full range of real-world judgments (refer to Saucier, 2003).

##### The Marker Scales for the English and Croatian Structures of Social Effects

We constructed marker scales for the two English social-effect factors based on the highest loading terms listed in Saucier (2010). A scale reflecting the extent to which a person is a source of pleasure to others, as well as a scale referring to the extent to which a person is a source of pain to others consisted of ten items each. Since markers were incorporated into the Lithuanian list of the social effects, the participants were also instructed to use the 7-point scale previously described. Reliability estimates for the first factor in self- and observer-rating data sets were α = 0.82 and α = 0.92, respectively, whereas for the second dimension the reliability estimates were α = 0.83 for self-ratings and α = 0.87 for observer-ratings.

The marker scales for the three Croatian social-effect factors were constructed based on the highest loading terms provided by Mlačić (2016). The scales measuring the Attractiveness-Popularity dimension and the Mysteriousness-Irritation factor included ten markers each, whereas the Likeability dimension was measured by eight items. For the Croatian marker scales, we used the same instruction as for the Lithuanian list of social effects. The reliability estimates for the three Croatian factors in the self- and observer-rating data sets were α = 0.85 and α = 0.88 (Attractiveness-Popularity), α = 0.68 and α = 0.78 (Mysteriousness-Irritation), and α = 0.76 and α = 0.86 (Likeability).

##### The Big Five Model Measure

We used Goldberg's (1992) IPIP-BFM-50, the 50-item Big Five Markers questionnaire from the resources of the International Personality Item Pool to measure the Big Five structure and to test the relations between the social-effect and personality structures. This questionnaire used a 5-point-scale (1 = strongly disagree to 5 = strongly agree). We translated IPIP-BFM-50 from English into Lithuanian for the purpose of the Lithuanian psycholexical project. The descriptive statistics and reliability estimates for IPIP-BFM-50 calculated based on the data collected in the current study are presented in Table 1.

**Table 1:** Descriptive statistics and reliability estimates for IPIP-BFM-50 and HEXACO-60.

	**Measure**	**Scale**	***N***	**α**	***M***	***SD***
Self-Ratings	IPIP-BFM-50	EXT	96	0.81	3.35	0.68
		AGR		0.89	3.70	0.70
		CON		0.71	3.37	0.63
		NEU		0.85	3.20	0.80
		INT		0.69	3.60	0.56
	HEXACO-60	EXT	107	0.76	3.19	00.61
		AGR		0.73	3.14	0.59
		CON		0.69	3.14	0.60
		EMO		0.63	3.17	0.58
		OPN		0.71	3.45	0.61
		H-H		0.78	3.11	0.65
Observer-Ratings	IPIP-BFM-50	EXT	109	0.88	3.38	0.95
		AGR		0.88	3.37	0.77
		CON		0.75	3.36	0.69
		NEU		0.85	3.00	0.75
		INT		0.81	3.36	0.72
	HEXACO-60	EXT	95	0.76	3.32	0.62
		AGR		0.83	2.94	0.75
		CON		0.83	3.00	0.76
		EMO		0.70	2.96	0.57
		OPN		0.82	2.92	0.78
		H-H		0.87	2.77	0.80

*EXT, Extraversion; AGR, Agreeableness; CON, Conscientiousness; NEU, Neuroticism; INT, Intellect; EMO, Emotionality; OPN, Openness; H-H, Honesty-Humility; N, number of participants; M, mean; SD, standard deviation; α, Cronbach's alpha coefficient*.

##### The HEXACO Model Measure

We used HEXACO-60 (Ashton and Lee, 2009) to measure the six-factor model of personality structure using a 5-point scale (1 = strongly disagree to 5 = strongly agree). Truskauskaitė-Kunevičienė et al. (2012) translated and culturally adapted HEXACO-60 for the Lithuanian population. Table 1 presents the descriptive statistics and reliability estimates for this measure based on the current study.

##### Participants and Procedures

We recruited two samples for this study, with one for observer-ratings and the other for self-ratings. The observer-ratings were provided by 207 students, of which three were excluded either because they provided incomplete responses or because they were not familiar with the meaning of at least 10% of the terms. The self-ratings were collected from 209 students, of which six were excluded for the same reasons as observer-ratings were removed. In the observer-rating sample (63.2% women, 36.3% men, 0.5% other), the age of the participants ranged from 18 to 48 years (mean age = 21.65, *SD* = 3.59), whereas in the self-rating sample (56.7% women, 42.9% men, 0.4% other) the age ranged from 18 to 29 years (mean age = 21.3, *SD* = 2.2). Most of the respondents were based in Vilnius and were enrolled in over 90 different majors at 15 universities or colleges in Lithuania.

It is worth noting that the ratio of participants (209/207) to variables (232) in the present study is not of concern, because the stability of a factor solution depends on the sampling error of the correlation coefficient, which decreases with the square root of the sample size and the absolute value of the loadings, regardless of the number of variables (refer to Guadagnoli and Velicer, 1988). Each of the two samples allows the detection of the population correlation coefficients |*r*| ≥ 0.2 with 90% power and α = 0.05 (one-side test).

The participants were recruited by three interviewers who contacted the students at their homes. The interviewers explained the purpose of the study and the instructions to every participant in face-to-face interaction, and the students had an opportunity to clarify any questions that arose. We asked each respondent to complete the Lithuanian list of social-effect descriptors, whereas approximately half of each sample filled out the HEXACO-60 questionnaire, and the second half—IPIP-BFM-50.

##### Data Analysis

Prior to qualitative data analysis, we pooled self-ratings with observer-ratings to assess the familiarity of terms, and eliminated 24 descriptors that were not clear, or were avoided by at least 10% of the participants. For all the social-effects descriptors, the range for skewness in the self-rating data [−1.23, 1.33] and observer-rating data [−0.82, 1.51] was acceptable. We ipsatized each participant's responses to a final set of 208 descriptors of social effects that were used in the analyses to remove individual differences in the rating scale used. The data for IPIP-BFM-50 and HEXACO-60 was not ipsatized. To obtain recommendations for the appropriate number of components, we applied parallel analysis (Horn, 1965) for the self-ratings and observer-ratings separately. The difference between self-rating and observer-rating structures was measured by using a principal component analysis for each type of data, and consequently computing Tucker's congruence coefficients between the self-rating and observer-rating varimax-rotated components. A congruence coefficient value in a range between 0.85 and 0.94 indicates that structures are fairly similar, whereas a congruence coefficient of 0.95 or higher suggests that structures are identical (Lorenzo-Seva and ten Berge, 2006).

We determined the optimal and most informative structures for each data set by evaluating the robustness of the components, regardless of the rotation method (refer to Saucier and Iurino, 2020). The decision on final structures in each data set was made based on the content analysis of the most robust solutions, as well as the number of terms with the highest magnitude loadings. The minimum number of terms with the highest magnitude loadings with an absolute value of more than 0.3 was determined to be six per dimension, provided at least one loading was above 0.5.

The relations between the Lithuanian, English, and Croatian social-effect structures, as well as between the Lithuanian social-effect components and personality dimensions were assessed by calculating the linear correlation coefficients. Although previous studies had not established a strict cut-off correlation coefficient value for declaring the replication of the cross-cultural factors, we took into consideration correlations of |*r*| > 0.5 (refer to Saucier, 2009; De Raad et al., 2010).

## Results

### Determining the Number of Components

The parallel analysis recommended 12 components for self-ratings, and six components for observer-ratings. To test the difference between the self-rating and observer-rating structures, we used a principal component analysis for each type of data and consequently computed Tucker's (1951) congruence coefficients between the self-rating and observer-rating varimax-rotated components from one to six-component solutions (refer to Table 2). The congruence coefficients between corresponding components were only ≥ 0.85 for the one and two-component solutions, therefore, the self-rating and observer-rating structures could be interpreted as at least similar only at these levels of hierarchy. Beginning with the three-component solution, most of the pairs of components failed to reach the level of 0.85, which indicated that the structures of the self-rating and observer-rating data sets were different and should be analyzed separately.

**Table 2:** Robustness indices.

***N*** **of factor**	**Tucker's phi coefficients (self- vs. observer-rating)**	**Orthogonal-Oblique best-match correlation**	
		**Self-Rating**	**Observer-Rating**
1	0.95	1,0	1.0
2	0.93, 0.91	0.98, 1.00	0.96, 1.00
3	0.86, 0.82, 0.44	0.96, 1.00, 0.99	0.94, 0.98, 1.00
4	0.89, 0.81, 0.90, 0.55	0.98, 1.00, 0.99, 0.97	0.96, 0.98, 0.99, 1.00
5	0.85, 0.79, 0.38, 0.89, 0.13	0.99, 1.00, 0.97, 0.98, 0.96	0.97, 0.95, 0.99, 0.98, 0.95
6	0.89, 0.82, 0.76, 0.56, 0.57, 0.26	0.97, 1.00, 0.96, 0.96, 0.79, 0.90	0.95, 0.98, 0.99, 0.97, 0.83, 0.96
7	–	0.97, 0.75, 0.75, 0.88, 0.86, 0.65, 0.94	–
8	–	0.93, 0.59, 0.77, 0.80, 0.58, 0.93, 0.91, 0.67	–
9	–	0.94, 1.00, 0.96, 0.97, 0.89, 0.97, 0.94, 0.85, 0.93	–
10	–	0.86, 1.00, 0.95, 0.64, 0.86, 0.96, 0.68, 0.63, 0.91, 0.78	–
11	–	0.87, 1.00, 0.97, 0.87, 0.94, 0.96, 0.90, 0.83, 0.92, 0.80, 0.89	–
12	–	0.82, 1.00, 0.98, 0.97, 0.94, 0.95, 0.75, 0.60, 0.93, 0.66, 0.89, 0.65	–

To determine the optimal and most informative structures for each data set, we evaluated the robustness of the components regardless of the rotation method. We calculated the correlations between the oblimin and varimax principal component structures for the self-ratings and observer-ratings separately (refer to Saucier and Iurino, 2020). The orthogonal-oblique best-match correlations are presented in Table 2. Taking 0.69 and the lower correlation coefficient as an insufficient degree of replication, the most robust structures for the self-rating data were the 1–6, 9, and 11-component structures, whereas for the observer-ratings all the components within the one to six-component solutions were replicated regardless of the type of rotation. Based on all the indices we had taken into account, including the number of terms with the highest magnitude loadings, the most robust and informative structures are most likely to be the five-factor solution for self-ratings and the four-factor solution for observer-ratings. To further investigate the lexicon of social effects and test our initial findings regarding the optimal structure for each perspective, we checked the interpretability of varimax-rotated components of the most robust solutions for self-ratings and observer-ratings separately.

### Observer-Rating Perspective

#### How Do Other People Affect Our Cognitive, Emotional, and Motivational Processes? (Emic Dimensions)

**Figure 1:**
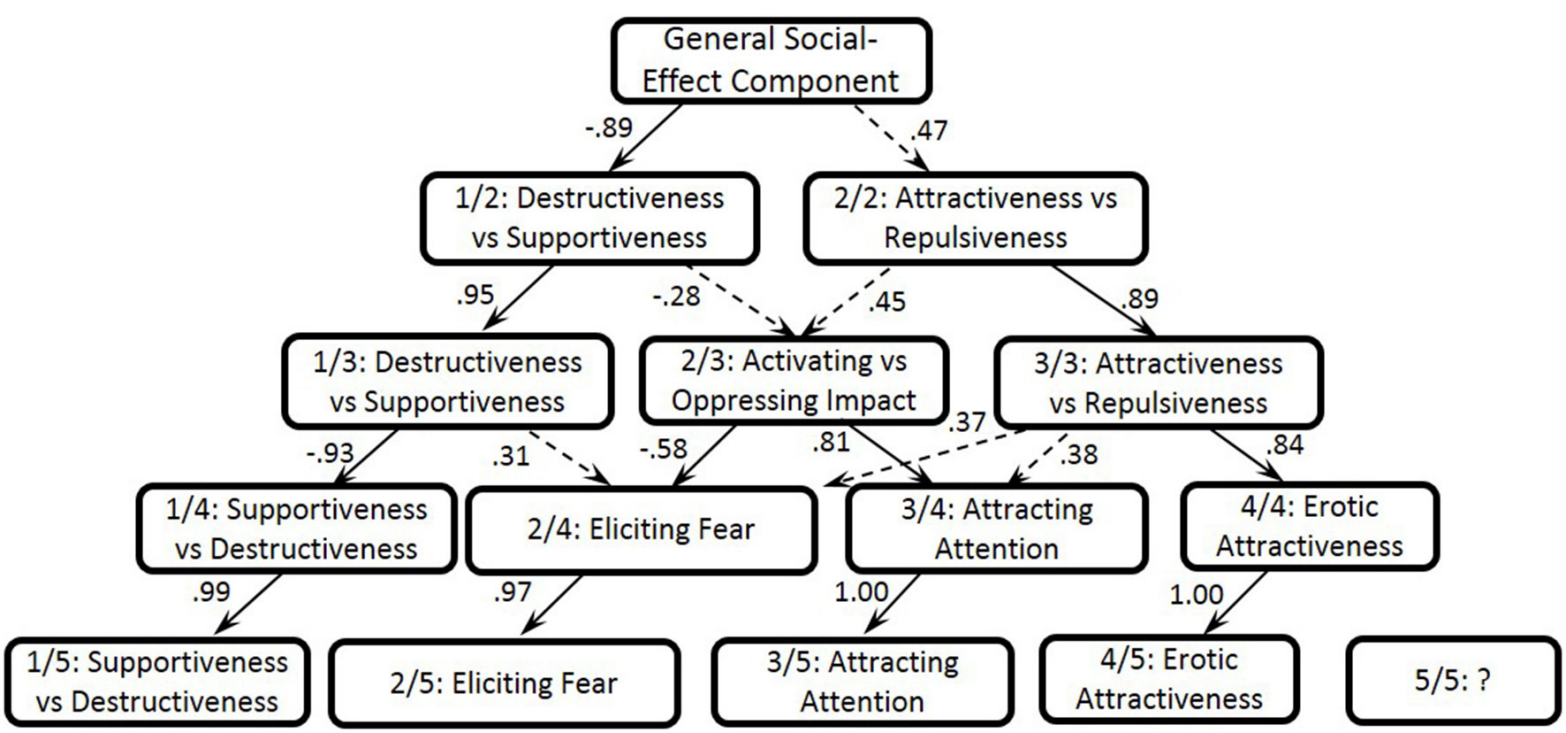
One to five-component hierarchical structure of the social effects based on the observer-rating data.

For the ipsatized observer-rating data matrix, the eigenvalues of the first 15 unrotated components for the 208 variables were 56, 10, 6.85, 5.99, 4.06, 3.56, 3.16, 3.05, 2.87, 2.84, 2.73, 2.61, 2.59, 2.42, and 2.33. The most evident elbow in the scree plot followed the second and fourth components. According to the standards of psycholexical studies, we examined the structures from the highest (one-component solution) to the lowest (six-component solution) levels, and the linear correlations between the dimensions from the previous and next levels of the hierarchy are presented in Figure 1 (Goldberg, 2006).

The first unrotated Lithuanian component explained 26.9% of the total variance and contrasted socially desirable and undesirable social effects. We labeled this dimension as General Social-Effect Component (1/1). The highest loading terms^2^ were encouraging (*padra̧sinantis*, 0.79), amazing (*nuostabus*, 0.77), lovable (*mielas*, 0.77), entertaining (*pralinksminantis*, 0.77), and supportive (*palaikantis*, 0.76) vs. unamiable (*nemalonus*, −0.74), irritating (*dirginantis*, −0.73), unlovable (*nemielas*, −0.72), unbearable (*nepakenčiamas*, −0.71), and exhausting (*išsekinantis*, −0.7).

At the two-component level, the General Social-Effect Component is split into two components (refer to Figure 1), the first of which described the Destructiveness vs. Supportiveness (1/2, 22.1% of the explained variance). The highest loading terms were encouraging (*padra̧sinantis*, −0.79), supportive (*palaikantis*, −0.77), calming (*nuraminantis*, −0.75), lovable (*mielas*, −0.75), trustworthy (*patikimas*, −0.74), and exhilarating (*pradžiuginantis*, −0.7) vs. irritating (*dirginantis*, 0.71), harmful (*kenkiantis*, 0.69), driving a wedge between somebody (*supriešinantis*, 0.68), abasing somebody (ž*eminantis kitus*, 0.66), and nerve-racking (*keliantis i̧tampa*, 0.66). The second component reflected Attractiveness vs. Repulsiveness (2/2, 5.9% of the explained variance), i.e., how attractive or repulsive other people are to us. High loading terms included enticing (*gundantis*, 0.58), impressive (*i̧spūdingas*, 0.57), alluring (*viliojantis*, 0.56), desirable (*geidžiamas*, 0.54), and exciting (*jaudinantis*, 0.53) vs. indistinct (*neryškus*, −0.7), repulsive (*neviliojantis*, −0.7), unsexy (*neseksualus*, −0.67), undesirable (*negeidžiamas*, −0.65), and boring (*nuobodus*, −0.57).

At the three-component level, the previous two components, namely Destructiveness vs. Supportiveness (1/3, 19.9% of the explained variance) and Attractiveness vs. Repulsiveness (3/3, 6.7% of the explained variance) were largely replicated with correlation coefficients of 0.95 and 0.89 with the respective higher-order components. At this level, a new dimension emerged, which described the Activating vs. Oppressing Impact that others have on us (2/3, 8.4% of the explained variance). The highest loading terms were frightful (*klaikus*, −0.54), scary (*baisus*, −0.53), horrible (š*iurpus*, −0.51), intimidating (*bauginantis*, −0.51), and hateful (*nekenčiamas*, −0.5) vs. making somebody speak (*sugebantis prakalbinti*, 0.57), attracting interest (*sugebantis sudominti*, 0.53), amusing (*sugebantis i̧linksminti*, 0.51), noticeable (*pastebimas*, 0.51), and enlivening (*pagyvinantis*, 0.5).

At the four-component level, the dimension labeled the Supportiveness vs. Destructiveness (1/4, 18.2% of the explained variance) was almost fully replicated as it had a correlation coefficient of 0.93 with the upper-level component. The remaining dimensions were at least partially split. The second emerging component was mainly associated with the emotions of anxiety and described as Eliciting Fear in observers (2/4, 7.1% of the explained variance): intimidating, scary, frightening, pernicious vs. likable, acceptable, unthreatening (refer to Table 3 for more detailed information). After splitting, the third component more strongly emphasized the cognitive aspect of our reaction to other people, that is, to what extent others Attract our Attention (3/4, 6.9% of the explained variance): distinct, noticeable, persuasive, memorable vs. indistinct, unnoticed, unknown, unrecognizable. Whereas, the fourth component mainly reflected the Erotic Attractiveness of other people (4/4) and how strong a reaction of desire they provoke in us. Thus, high loading terms included enticing, impassioning, desirable, erotic, sexy, mysterious, hypnotizing vs. repulsive, unsexy, unattractive. The four-component solution explained 37.9% of the total variance.

**Table 3:** Varimax-rotated four-factor structure of the social effects in the observer-rating data (20 highest loading terms for each dimension).

	**Principal components**				
**Lithuanian term**	**1**	**2**	**3**	**4**	**English translation**
nuraminantis	**0.76**	−0.20	0.03	0.07	Calming
padra̧sinantis	**0.72**	**−0.33**	0.14	0.12	Encouraging sb
pykdantis	**−0.70**	0.10	−0.12	−0.18	Making sb angry
motyvuojantis	**0.70**	−0.07	**0.31**	0.05	Motivating
suteikiantis stiprybės	**0.69**	−0.10	0.26	0.17	Heartening
palaikantis	**0.69**	**−0.36**	0.15	0.07	Supportive
patikimas	**0.69**	−0.29	0.08	0.01	Trustworthy
nuostabus	**0.68**	−0.23	0.19	0.25	Amazing
dirginantis	**−0.68**	0.24	−0.15	−0.16	Irritating
paguodžiantis	**0.67**	−0.26	0.07	0.11	Comforting
siutinantis	**−0.67**	0.07	−0.12	−0.15	Enraging
praturtinantis kitus	**0.66**	−0.11	0.18	0.01	Enriching
nervinantis	**−0.66**	−0.11	−0.16	**−0.31**	Making sb nervous
sukeliantis kitiems laimȩ	**0.66**	−0.21	0.07	0.05	Elating
mielas	**0.66**	**−0.38**	0.07	0.24	Lovable
branginamas	**0.65**	−0.18	0.09	0.19	Appreciated
i̧šsekinantis	**−0.65**	0.11	−0.26	−0.18	Exhausting
i̧kvepiantis	**0.65**	−0.08	0.20	0.14	Inspiring
apgaulingas	**−0.64**	0.05	−0.16	−0.05	Deceptive
nevarginantis	**0.64**	−0.24	0.07	−0.04	Not tiring
…	…	…	…	…	…
bauginantis	−0.03	**0.68**	−0.15	−0.01	Intimidating
šiurpus	−0.19	**0.64**	−0.19	−0.01	Horrible
baisus	−0.15	**0.62**	−0.25	−0.22	Scary
kraupus	−0.25	**0.59**	−0.21	−0.21	Terrifying
klaikus	−0.21	**0.57**	−0.29	−0.13	Frightful
siaubingas	**−0.35**	**0.56**	−0.24	−0.19	Terrible
keliantis pasibaisejima̧	**−0.36**	**0.54**	−0.26	−0.18	Dreadful
šlykštus	**−0.42**	**0.54**	−0.25	−0.17	Abominable
pakenčiamas	**0.31**	**−0.54**	−0.02	0.02	Bearable
ga̧sdinantis	−0.09	**0.52**	−0.04	−0.05	Frightening
sugebantis itikti	0.03	**−0.51**	0.08	0.04	Pleasing
patinkantis kitiems	**0.31**	**−0.51**	0.27	0.22	Likeable
pražūtingas	−0.26	**0.51**	−0.15	−0.10	Pernicious
priimtinas kitiems	**0.40**	**−0.50**	0.12	0.00	Acceptable
skriaudžiantis	**−0.33**	**0.49**	−0.08	−0.11	Harmful
pravirkdantis	−0.17	**0.49**	0.03	0.06	Making sb cry
grėsmingas	−0.12	**0.47**	−0.01	−0.08	Threatening
pavojingas	−0.22	**0.46**	−0.07	0.10	Dangerous
apgailetinas	**−0.40**	**0.44**	−0.25	−0.28	Pathetic
atstumiantis	**−0.39**	**0.43**	−0.27	−0.21	Repulsive
…	…	…	…	…	…
neryškus	0.03	−0.05	**−0.69**	**−0.30**	Indistinct
nematomas	0.10	−0.07	**−0.63**	−0.11	Unnoticed
nepastebimas	0.09	−0.16	**−0.63**	−0.11	Unnoticeable
neišraiškingas	−0.07	−0.10	**−0.58**	−0.21	Inexpressive
nuobodus	−0.31	0.04	**−0.58**	−0.21	Boring
nežinomas	−0.06	−0.06	**−0.58**	−0.05	Unknown
nei̧domus	**−0.34**	0.15	**−0.58**	−0.21	Uninteresting
išraiškingas	0.20	−0.13	**0.53**	0.13	Expressive
ryškus	0.00	−0.01	**0.52**	0.16	Distinct
pastebimas	0.16	−0.18	**0.52**	0.08	Noticeable
nesugebantis i̧tikinti	−0.16	−0.03	**−0.48**	−0.11	Not able to convince sb
neefektingas	−0.28	−0.11	**−0.48**	−0.20	Inconspicuous
matomas	0.06	−0.19	**0.48**	−0.02	Noticed
negeidžiamas	−0.08	0.17	**−0.47**	**−0.46**	Undesirable
i̧simintinas	−0.01	−0.10	**0.47**	0.15	Memorable
pagyvinantis	**0.40**	−0.24	**0.47**	−0.02	Enlivening
sugebantis i̧kalbeti	0.20	−0.26	**0.45**	0.01	Persuasive
neatpažistamas	−0.13	0.18	**−0.44**	−0.01	Unrecognizable
sugebantis i̧tikinti	0.24	−0.22	**0.42**	0.10	Able to convince sb
žinomas	0.08	−0.09	**0.42**	−0.04	Famous
…	…	…	…	…	…
gundantis	0.15	−0.17	0.11	**0.72**	Enticing
viliojantis	0.16	−0.19	0.15	**0.67**	Alluring
sukeliantis aistra̧	0.21	−0.08	0.04	**0.66**	Impassioning
erotiškas	0.19	0.02	−0.01	**0.65**	Erotic
seksualus	0.20	−0.14	0.09	**0.64**	Sexy
neseksualus	−0.04	0.10	**−0.34**	**−0.63**	Not sexy
jaudinantis	0.25	−0.02	0.12	**0.62**	Exciting
geidžiamas	0.26	−0.21	0.17	**0.60**	Desirable
kerintis	**0.41**	−0.03	0.17	**0.59**	Charming
neviliojantis	−0.07	−0.03	**−0.40**	**−0.58**	Repulsive
patrauklus	**0.30**	−0.33	0.20	**0.58**	Attractive
nesimpatiškas	**−0.30**	0.22	−0.27	**−0.57**	Unattractive
žavus	**0.44**	−0.31	0.22	**0.53**	Alluring
hipnotizuojantis	0.07	0.18	0.16	**0.51**	Hypnotizing
nepatrauklus	−0.27	0.28	−0.41	**−0.49**	Unattractive
simpatiškas	**0.39**	**−0.42**	0.17	**0.48**	Attractive
pribloškiantis	0.26	0.13	0.20	**0.43**	Stunning
pavergiantis	0.16	0.15	0.16	**0.43**	Irresistible
pritrenkiantis	**0.39**	0.09	0.26	**0.43**	Stunning
saldus	−0.14	−0.24	−0.14	**0.43**	Sweet
…	…	…	…	…	…

*Loadings with absolute values of 0.3 or greater are given in bold type*.

A more detailed analysis of the social-effect types showed that emotional reactions were prevailing in the first and the second components (65 and 80% of the 20 highest loading terms, respectively), whereas the cognitive component was predominant in the third dimension (80% of the 20 highest loading terms). In the fourth component, motivational and emotional reactions appeared in the same proportion (40% of the 20 highest loading terms each), and the cognitive component constituted 25% of the highest loading terms. The percentage share of the different types of social effects appeared to be compatible with the content of the observer-rating components.

At the five- and six-component levels, all the dimensions from the four-component solution were fully replicated (refer to Figure 1) without changing their order, and, as at previous levels of the hierarchy, formed interpretable bipolar dimensions. In turn, new components explained only 2–2.5% of the total variance and were rather small low-saturation dimensions that included only 4–5 items with an absolute loading of 0.3–0.42. Additionally, these new components could not be interpreted unequivocally. All in all, based on the interpretability and saturation of components, as well as the robustness indices and the screen test, the four-component solution should be considered the most detailed and interpretable structure of social effects for the observer-rating data.

#### Social Effects as a Consequence of Perceived Personality Dispositions

To identify possible relations between our social-effects components and personality dimensions, we calculated the linear correlation coefficients between the component scores of the one to four-component solutions and the personality dimensions measured by IPIP-BFM-50 and HEXACO-60 (refer to Table 4). Here, we will only discuss in detail the relations between the most informative four-component social-effect structure and personality dimensions.

**Table 4:** Social effects upon perceived personality traits.

		**IPIP–BFM (*****N*** **=** **109)**					**HEXACO (*****N*** **=** **95)**					
**S/C**	**Lithuanian social-effect components**	**EXT**	**AGR**	**CON**	**NEU**	**INT**	**EXT**	**AGR**	**CON**	**EMO**	**OPN**	**HON**
1/1	General social-effect component	0.37	**0.76**	0.30	−0.36	**0.65**	0.21	**0.59**	0.42	0.05	0.39	**0.58**
1/2	Destructiveness vs. supportiveness	−0.12	**−0.66**	−0.22	0.26	**−0.55**	−0.05	**−0.53**	−0.38	−0.10	−0.27	**−0.60**
2/2	Attractiveness vs. repulsiveness	**0.54**	0.41	0.24	−0.29	0.37	0.38	0.22	0.16	−0.12	0.31	0.06
1/3	Destructiveness vs. supportiveness	0.02	**−0.60**	−0.19	0.20	**−0.50**	0.05	**−0.59**	−0.39	−0.10	−0.27	**−0.67**
2/3	Activating vs. oppressing Impact	**0.63**	0.43	0.20	−0.32	0.36	0.44	−0.01	0.08	−0.02	0.16	−0.08
3/3	Attractiveness vs. repulsiveness	0.32	0.29	0.18	−0.19	0.28	0.17	0.26	0.13	−0.11	0.24	0.12
1/4	Supportiveness vs. destructiveness	0.04	**0.59**	0.21	−0.26	**0.55**	0.00	**0.56**	0.39	0.15	0.33	**0.71**
2/4	Eliciting fear	−0.14	−0.32	−0.08	0.04	−0.15	−0.07	−0.10	−0.08	0.06	0.03	0.03
3/4	Attracting attention	**0.67**	0.37	0.21	−0.37	0.37	**0.52**	−0.03	0.08	0.00	0.27	−0.03
4/4	Erotic attractiveness	0.13	0.24	0.13	−0.04	0.15	0.01	0.28	0.11	−0.17	0.10	0.05

*Correlation coefficients with absolute value higher than 0.26 are statistically significant at p < 0.01. Coefficient with absolute values of 0.5 or greater are given in bold type. S/C, solution/component; EXT, Extraversion; AGR, Agreeableness; CON, Conscientiousness; NEU, Neuroticism; INT, Intellect; EMO, Emotionality; OPN, Openness; H-H, Honesty-Humility*.

Relatively stronger correlations were only observed for two social-effect dimensions. Thus, the social-effect component of Supportiveness vs. Destructiveness was most strongly related to two Big Five factors: Agreeableness (*r* = 0.59, *p* < 0.001) and Intellect (*r* = 0.55, *p* < 0.001) and three HEXACO factors: Honesty (*r* = 0.71, *p* < 0.001), Agreeableness (*r* = 0.56, *p* < 0.001), and Conscientiousness (*r* = 0.39, *p* < 0.001). Also, the social-effect factor of Attracting Attention was strongly related to one Big Five factor, Extraversion (*r* = 0.67, *p* < 0.001), and one HEXACO factor, also Extraversion (*r* = 0.52, *p* < 0.001). In turn, two other social-effect dimensions cannot be fully explained by the personality dispositions of the observed people. Persons who elicit fear in observers are perceived as less agreeable (*r* = −0.32, *p* < 0.001) in the context of IPIP-BFM-50, whereas in the case of HEXACO-60 no statistically significant correlations between this social-effect component and personality dispositions are observed. Furthermore, the social-effect factor of Erotic Attractiveness is poorly related to either IPIP-BFM-50 or HEXACO-60, with the largest correlation between that factor and Agreeableness from both instruments being 0.24 (*p* < 0.05) and 0.28 (*p* < 0.01), respectively.

#### Lithuanian Observer-Rating Social-Effect Dimensions Compared With the English and Croatian Social-Effect Lexicon Structures

To assess to what extent the structure of the Lithuanian lexicon of social effects is convergent with the respective English and Croatian lexicons, we calculated the linear coefficient of the correlations between the component scores for each Lithuanian solution containing from one to four components and the marker scales for the English and Croatian social-effect lexicons (refer to Table 5).

**Table 5:** The relationship between Lithuanian, English, and Croatian social-effect lexicons in the observer-rating data.

		**English SE lexicon**		**Croatian SE lexicon**		
**S/C**	**Lithuanian social-effect components**	**E1**	**E2**	**C1**	**C2**	**C3**
**Varimax-Rotated components**						
1/1	General social-effect component	**0.86**	**−0.82**	**0.64**	**−0.71**	**0.91**
1/2	Destructiveness vs. supportiveness	**−0.62**	**0.86**	−0.28	**0.80**	**−0.80**
2/2	Attractiveness vs. repulsiveness	**0.67**	−0.12	**0.84**	0.00	0.42
1/3	Destructiveness vs. supportiveness	**−0.61**	**0.81**	−0.27	**0.73**	**−0.75**
2/3	Activating vs. oppressing impact	0.37	−0.29	0.39	−0.31	0.41
3/3	Attractiveness vs. repulsiveness	**0.59**	−0.02	**0.76**	0.13	0.29
1/4	Supportiveness vs. destructiveness	**0.59**	**−0.71**	0.28	**−0.67**	**0.68**
2/4	Eliciting fear	−0.24	**0.50**	−0.10	0.42	−0.44
3/4	Attracting attention	0.39	−0.06	**0.51**	−0.10	0.27
4/4	Erotic attractiveness	**0.55**	−0.12	**0.67**	0.10	0.33
**Rerotated orthogonal components**						
1/2′	Destructiveness vs. supportiveness	−0.37	**0.77**	**–**	–	–
2/2′	Attractiveness vs. repulsiveness	**0.84**	−0.39	**–**	–	–

*N = 204. Correlation coefficients with absolute values higher than 0.19 are statistically significant at p < 0.01. Coefficients with absolute values of 0.5 or greater are given in bold type. S/C, solution/component; SE lexicon, social-effect lexicon; E1, the extent to which a person is a source of pleasure to others; E2, the extent to which a person is a source of pain to others; C1, Attractiveness-Popularity; C2, Mysteriousness vs. Irritation; C3, Likeability*.

The correlation coefficients between the Lithuanian two-component observer-rating social-effect structure and the respective English two-factor structure indicated that there were some differences in axis rotation in two-dimensional space. To be able to compare these two structures, we opted for re-rotating the axes of the Lithuanian social-effect dimensions to achieve the highest level of convergence between corresponding pairs of components, as well as the highest level of discrimination—the lowest correlation coefficients with other non-corresponding dimensions. This effect could be achieved by re-rotating both Lithuanian axes by 19 degrees (clockwise) while maintaining the orthogonality of the dimensions. Re-alignment by this angle did not substantially change the interpretation of the components (Refer to Appendix 1). The relations presented in Table 5 indicate that there is a high level of convergence between the Lithuanian and English social-effect components. The English factor describing the extent to which a person is a source of pleasure to others strictly correlates with the re-rotated Lithuanian dimension presenting Attractiveness vs. Repulsiveness (*r* = 0.84). In turn, the second English factor describing the extent to which a person is a source of pain to others highly correlates with the re-aligned Lithuanian dimension presenting the Destructiveness vs. Supportiveness of others (*r* = 0.77). The analysis of the lower-order structures points to the fact that the Lithuanian component of Attracting Attention has the weakest relations with both English social-effect factors.

The comparison of the Lithuanian and Croatian three-component solutions (refer to Table 5) shows the highest similarity between the Lithuanian Attractiveness vs. Repulsiveness and the Croatian Attractiveness-Popularity (*r* = 0.76). In turn, the Lithuanian dimension describing the Destructiveness vs. Supportiveness of others is reflected by two Croatian dimensions, namely Mysteriousness vs. Irritation (*r* = 0.73) and Likeability (*r* = −0.75). Whereas, the Lithuanian component presenting the Activating vs. Oppressing Impact has relatively low relations with all three Croatian social-effect dimensions—Likeability (*r* = 0.41), Attractiveness-Popularity (*r* = 0.39), and Mysteriousness vs. Irritation (*r* = 0.31). Similarly, Eliciting Fear in others from the Lithuanian four-component solution that emerges from the Activating vs. Oppressing Impact is not strongly related to the Croatian social-effect structure. It is worth noting that the re-rotation of any pair of Lithuanian factors does not significantly increase the convergence and divergence coefficients between the Lithuanian and Croatian social-effect structures.

### Self-Rating Perspective

#### How Do We Affect the Cognitive, Emotional, and Motivational Processes of Others? (Emic Dimensions)

**Figure 2:**
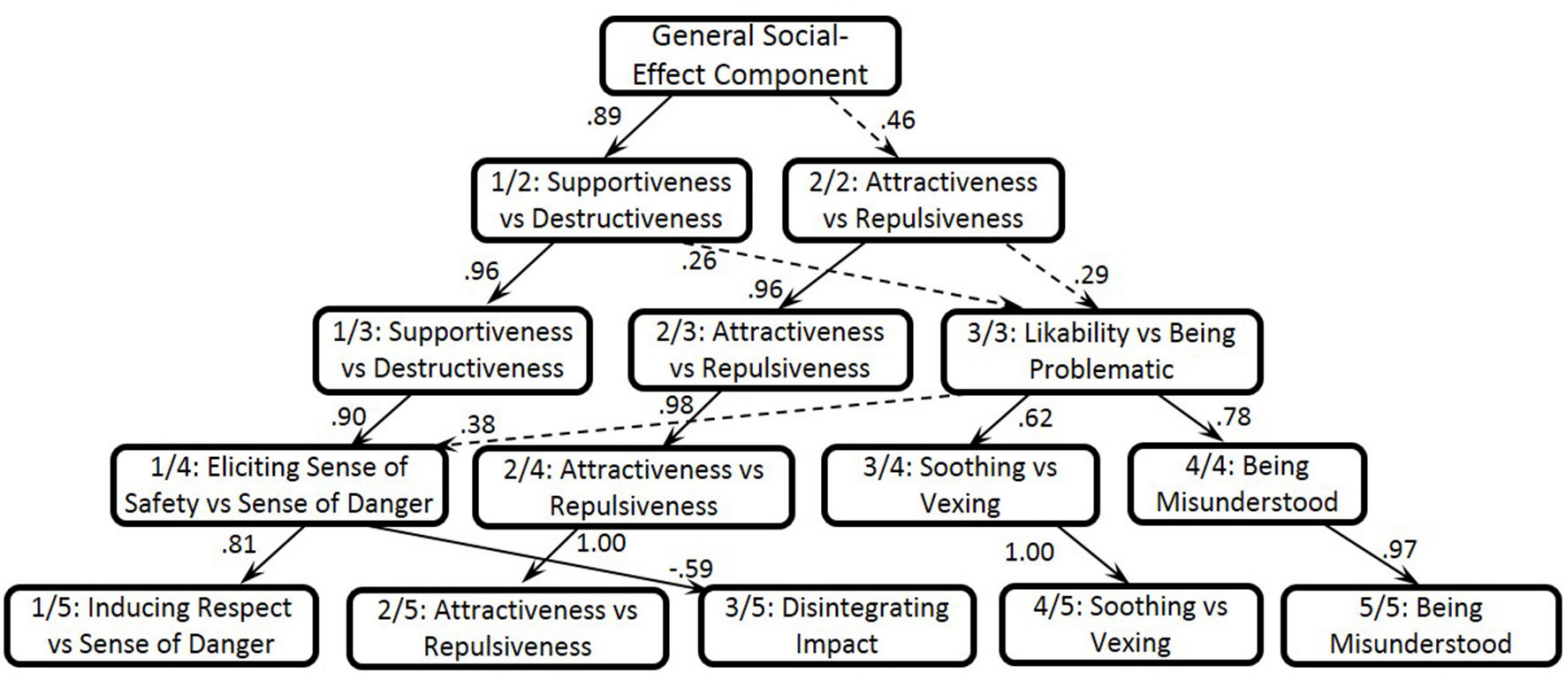
One to five-component hierarchical structure of the social effects based on the self-rating data.

For the ipsatized self-rating data matrix, the eigenvalues of the first 15 unrotated components for the 208 variables were 33.16, 12.65, 5.84, 4.6, 4.15, 4.02, 3.72, 3.47, 3.36, 3.2, 3.12, 3.06, 2.96, 2.91, and 2.82. Since the 1–6, 9, and 11-component structures were the most robust based on the orthogonal-oblique best-match correlations, we examined these structures beginning with the highest (one-component solution) to the lowest levels of the hierarchy (refer to Figure 2).

The first unrotated Lithuanian component explained 15.90% of the total variance and contrasted socially desirable and undesirable social effects. The highest loading terms for the General Social-Effect Component (1/1) were acceptable (*priimtinas kitiems*, 0.63), attractive (*patrauklus*, 0.62), amusing (*sugebantis i̧linksminti*, 0.6), likable (*patinkantis kitiems*, 0.59), and trustworthy (*patikimas*, 0.59) vs. dreadful (*keliantis pasibaisėjima*, −0.63), making somebody gloomy (*niūrinantis*, −0.58), abominable (š*lykštus*, −0.58), hateful (*nekenčiamas*, −0.58), frightening (*ga̧sdinantis*, −0.56), pathetic (*apgailėtinas*, −0.55), harmful (*skriaudžiantis*, −0.55), and enraging (*siutinantis*, −0.55).

At the two-component level, the General Social-Effect Component is divided into two components (refer to Figure 2), namely the Supportiveness vs. Destructiveness (1/2, 13.8% of the total variance) and Attractiveness vs. Repulsiveness (2/2, 8.2% of the total variance). The first dimension was described as trustworthy (*patikimas*, 0.66), encouraging (*paraginantis*, 0.62), calming (*nuraminantis*,0.62), supportive (*palaikantis*, 0.54), and motivating (*motyvuojantis*, 0.54) vs. frightening (*ga̧sdinantis*, −0.59), horrible (š*iurpus*, −0.58), enraging (*siutinantis*, −0.58), and harmful (ž*alojantis kitus*, −0.58). The second component was defined by terms such as enticing (*gundantis*, 0.66), sexy (*seksualus*, 0.63), desirable (*geidžiamas*, 0.62), stunning (*pritrenkiantis*, 0.61), impassioning (*sukeliantis aistra*, 0.58), attractive (*patrauklus*, 0.54), and noticeable (*pastebimas*, 0.53) vs. unsexy (*neseksualus*, −0.7), undesirable (*negeidžiamas*, −0.68), repulsive (*neviliojantis*, −0.68), unattractive (*nesimpatiškas*, −0.67), unattractive (*nepatrauklus*, −0.66), and indistinct (*neryškus*, −0.63).

In the three-component solution, two dimensions from the previous level stayed almost intact. The Supportiveness vs. Destructiveness (1/3, 12.9% of the total variance) and Attractiveness vs. Repulsiveness (2/3, 7.3% of the total variance) correlated at 0.96 and 0.96 with respect to the higher-order components. Additionally, a new component representing Likability vs. Being Problematic to others (3/3, 4.6% of the total variance) appeared, and included such attributes as lovable (*mielas*, 0.51), charming (*kerintis*, 0.46), coherent (*suprantamas*, 0.43), clear (*aiškus*, 0.38), amazing (*nuostabus*, 0.36), and softening somebody (*sušvelninantis kitus*, 0.34) vs. complicated (*komplikuotas*, −0.45), unclear (*neaiškus*, −0.45), offensive (*sugebantis užgauti*, −0.43), annoying (*i̧kyrus*, −0.42), misunderstood (*nesuprantamas*, −0.42), and problematic (*keblus*, −0.4).

At the four-component level, only Attractiveness vs. Repulsiveness (2/4, 5.9% of the total variance) remained. Other components from the previous solution were at least partially split. The first dimension described Eliciting a Sense of Safety vs. a Sense of Danger in others (1/4, 10.7% of the total variance) with the highest loading terms of trustworthy (*patikimas*, 0.59), acceptable (*priimtinas kitiems*,0.57), encouraging somebody (*paraginantis*, 0.56), entertaining (*pralinksminantis*, 0.52), useful (*naudingas*, 0.52), unthreatening (*negrėsmingas*, 0.51), and harmless (*nekenksmingas*, 0.46) vs. horrible (š*iurpus*, −0.65), dreadful (*keliantis pasibaisėjima*, −0.62), abominable (š*lykštus*, −0.6), frightful (*klaikus*, −0.6), harmful (ž*alojantis kitus*, −0.6), and intimidating (*bauginantis*, −0.59). The third component from the upper-level solution split into two new dimensions, of which the first one described the Soothing vs. Vexing effect (3/4, 5.9% of the total variance) defined by not causing stress (*nesukeliantis i̧tampos kitiems*, 0.52), not tiring (*nevarginantis*, 0.52), calming (*nuraminantis*, 0.46), enriching (*praturtinantis kitus*, 0.44), and softening (*sušvelninantis kitus*, 0.38) vs. insulting (*ižeidžiantis*, −0.43), causing pain (*skaudinantis*, −0.42), tiresome (*varginantis*, −0.41), exhausting (*išsekinantis*, −0.4), nerve-racking (*keliantis i̧tampa*, −0.38), and confusing (*klaidinantis*, −0.37). The second component mainly reflected Being Misunderstood by others (4/4, 3.2% of the total variance) described as unclear (*neaiškus*, −0.54), bizarre (*keistas*, −0.5), intricate (*sudėtingas*, −0.47), complicated (*komplikuotas*, −0.46), misunderstood (*nesuprantamas*, −0.44), problematic (*keblus*, −0.37), embarrassing (*gluminantis*, −0.34), and enigmatic (*mi̧slingas*, −0.3).

In the five-component solution, the second, fourth, and fifth dimensions replicated the second, third, and fourth upper-level components, respectively, with the correlation coefficients ranging from 0.97 to 1. In turn, the first upper-level component is split into two dimensions, of which one reflected Inducing Respect vs. a Sense of Danger in others (1/5), and the second represents Inducing a Disintegrating vs. Exhilarating/Activating/Integrating Impact on others (3/5) (refer to Figure 2). To enhance our analysis, we used principal component analysis to scrutinize the highest loading terms related to each component and were able to distinguish subcomponents for each dimension at the five-component level (refer to Table 6).

**Table 6:** Varimax-rotated five-factor structure of the social effects in the self-rating data (20 highest loading terms for each dimension).

	**Principal components**					
**Lithuanian term**	**1**	**2**	**3**	**4**	**5**	**English translation**
šlykštus	**−0.63**	−0.17	0.18	−0.05	−0.03	Abominable
žalojantis kitus	**−0.61**	−0.06	0.22	−0.09	0.18	Harmful
patikimas	**0.59**	−0.03	−0.23	0.26	0.00	Trustworthy
padra̧sinantis	**0.57**	−0.03	0.00	**0.38**	0.13	Encouraging
naudingas	**0.56**	0.05	−0.15	0.24	−0.05	Useful
siaubingas	**−0.55**	−0.20	0.12	−0.14	−0.14	Terrible
kraupus	**−0.55**	−0.16	0.25	−0.11	−0.07	Terrifying
paraginantis	**0.55**	−0.11	−0.24	0.22	−0.03	Encouraging
branginamas	**0.54**	0.15	0.07	0.21	0.22	Appreciated
šiurpus	**−0.52**	−0.08	**0.40**	−0.03	−0.08	Horrible
skriaudžiantis	**−0.50**	−0.08	**0.33**	−0.13	0.08	Harmful
skatinantis kitus	**0.49**	−0.14	−0.20	0.21	−0.11	Encouraging
pražūtingas	**−0.48**	−0.04	0.14	−0.15	−0.05	Pernicious
brangus kitiems	**0.47**	0.09	−0.07	0.18	0.27	Appreciated
sugebantis palenkti kitus kažkam	**0.47**	0.14	0.00	0.03	−0.06	Able to incline sb
paprotinantis kitus	**0.46**	−0.02	0.12	0.17	0.02	Instructing
klaikus	**−0.46**	−0.26	**0.41**	0.04	0.06	Frightful
bjaurus	**−0.45**	−0.18	0.17	−0.11	−0.11	Disgusting
keliantis nerima̧	**−0.44**	−0.17	0.19	−0.14	−0.10	Disturbing
grėsmingas	**−0.44**	−0.08	0.40	−0.08	−0.05	Threatening
…	…	…	…	…	…	…
neviliojantis	0.05	**−0.69**	0.03	0.07	−0.08	Repulsive
neryškus	−0.09	**−0.69**	0.03	0.00	0.11	Indistinct
nepastebimas	−0.19	**−0.65**	0.00	0.06	0.11	Unnoticeable
neseksualus	0.03	**−0.64**	0.21	−0.03	−0.27	Not sexy
nesimpatiškas	−0.13	**−0.63**	0.00	−0.02	−0.23	Unattractive
nepatrauklus	−0.23	**−0.62**	0.17	−0.03	−0.17	Unattractive
negeidžiamas	−0.14	**−0.62**	0.09	−0.02	−0.27	Undesirable
nei̧domus	−0.31	**−0.60**	0.14	−0.13	0.19	Uninteresting
nuobodus	−0.24	**−0.60**	0.00	0.01	0.17	Boring
gundantis	−0.03	**0.57**	−0.01	0.08	**0.38**	Enticing
pritrenkiantis	0.11	**0.57**	0.04	0.05	0.23	Stunning
pribloškiantis	−0.13	**0.56**	−0.02	0.17	0.10	Stunning
stulbinantis	−0.01	**0.55**	−0.04	−0.01	0.12	Stunning
geidžiamas	0.04	**0.55**	−0.18	0.08	0.28	Desirable
pritraukiantis	0.05	**0.55**	−0.07	0.21	0.02	Attractive
seksualus	−0.13	**0.53**	−0.09	0.08	**0.39**	Sexy
i̧spūdingas	−0.03	**0.53**	−0.11	0.00	0.11	Impressive
pastebimas	0.08	**0.51**	0.12	0.11	0.01	Noticeable
ryškus	−0.02	**0.50**	0.10	0.05	−0.13	Distinct
viliojantis	−0.17	**0.49**	−0.17	−0.08	0.30	Alluring
…	…	…	…	…	…	…
keliantis pasibaisejima̧	**−0.39**	−0.28	**0.54**	−0.04	0.01	Dreadful
pamaloninantis	0.05	0.00	**−0.54**	0.13	0.16	Pleasing
sugebantis prajuokinti	0.22	0.14	**−0.52**	0.14	−0.17	Making sb laugh
megiamas	0.25	0.15	**−0.47**	0.12	0.18	Likeable
sugebantis i̧linksminti	0.27	0.27	**−0.46**	0.22	0.05	Amusing
malonus	**0.41**	−0.08	**−0.45**	0.26	0.13	Amiable
išsekinantis	−0.07	−0.12	**0.45**	**−0.38**	0.00	Exhausting
sugebantis i̧siteikti	−0.01	0.05	**−0.45**	0.00	−0.07	Worming oneself into sb's good graces
niūrinantis	**−0.38**	−0.18	**0.44**	−0.14	0.00	Making sb gloomy
dirginantis	−0.26	−0.02	**0.44**	−0.08	−0.03	Irritating
ga̧sdinantis	**−0.41**	−0.02	**0.44**	−0.15	−0.12	Frightening
bauginantis	**−0.43**	−0.13	**0.43**	−0.06	−0.06	Intimidating
pralinksminantis	**0.35**	0.07	**−0.43**	0.12	0.01	Entertaining
nekenčiamas	**−0.38**	−0.16	**0.42**	−0.15	−0.08	Hateful
nemalonus	−0.24	−0.15	**0.42**	−0.23	0.00	Unamiable
nukankinantis	**−0.39**	−0.05	**0.42**	−0.11	−0.01	Overtiring
priimtinas kitiems	**0.42**	0.11	**−0.42**	0.23	0.17	Acceptable
sugebantis i̧sigerinti	0.06	0.06	**−0.40**	−0.15	0.07	Worming oneself into sb's favour
pagyvinantis	−0.02	0.14	**−0.40**	0.16	0.05	Enlivening
siutinantis	**−0.34**	−0.06	**0.39**	**−0.30**	0.09	Enraging
…	…	…	…	…	…	…
nesukeliantis i̧tampos kitiems	0.25	−0.25	−0.14	**0.51**	0.05	Not causing stress to sb
nevarginantis	0.25	−0.04	−0.17	**0.50**	0.13	Not tiring
erzinantis	−0.10	−0.18	0.02	**−0.48**	0.01	Irritating
i̧grystantis	−0.12	−0.25	0.01	**−0.48**	−0.10	Pestering sb
i̧kyrus	0.01	−0.10	−0.04	**−0.46**	−0.16	Annoying
sugebantis sukelti i̧nirši̧	−0.01	0.10	0.10	**−0.46**	0.03	Making sb furious
trikdantis	−0.15	−0.10	0.20	**−0.46**	−0.11	Troublesome
sugebantis užgauti	0.06	−0.10	0.03	**−0.45**	−0.16	Offensive
nervinantis	−0.07	−0.24	0.05	**−0.44**	−0.06	Making sb nervous
sugebantis sukelti i̧tūži̧	−0.04	−0.02	0.21	**−0.44**	0.12	Making sb furious
trukdantis	−0.20	−0.26	0.06	**−0.43**	−0.06	Disturbing
nuraminantis	**0.34**	−0.02	**−0.33**	**0.43**	0.03	Calming
praturtinantis kitus	**0.32**	0.07	−0.07	**0.42**	−0.05	Enriching
i̧žeidžiantis	−0.11	−0.10	**0.30**	**−0.41**	0.05	Insulting
skaudinantis	−0.17	−0.12	0.29	**−0.40**	−0.16	Causing pain
sugebantis “supjudyti”	−0.19	−0.04	**0.36**	**−0.40**	0.10	Setting others at variance
varginantis	−0.11	−0.17	**0.39**	**−0.39**	0.11	Tiresome
aiškus	0.21	0.02	0.05	**0.39**	0.23	Clear
kvaršintojas	−0.02	−0.07	−0.01	**−0.37**	−0.08	Bothering
keliantis i̧tampa̧	−0.17	0.05	0.29	**−0.36**	−0.11	Nerve-racking
…	…	…	…	…	…	…
neaiškus	−0.17	−0.12	−0.07	−0.06	**−0.59**	Unclear
keistas	0.00	−0.17	−0.08	0.15	**−0.52**	Bizarre
mielas	0.20	0.11	**−0.35**	0.24	**0.47**	Lovable
komplikuotas	0.09	−0.14	−0.07	−0.16	**−0.45**	Complicated
sudėtingas	0.28	0.00	−0.08	−0.08	**−0.45**	Intricate
simpatiškas	0.29	**0.34**	−0.19	0.07	**0.40**	Attractive
nuostabus	0.16	**0.33**	0.11	0.13	**0.40**	Amazing
keblus	−0.16	−0.08	0.07	−0.20	**−0.39**	Problematic
nesuprantamas	0.11	−0.28	0.19	−0.14	**−0.38**	Misunderstood
atbaidantis kitus	−0.26	−0.17	0.12	−0.19	**−0.35**	Scary
gluminantis	−0.10	−0.11	0.03	−0.26	**−0.35**	Embarrassing
saldus	−0.09	0.27	−0.29	0.03	**0.33**	Sweet
mi̧slingas	0.07	0.10	0.04	0.08	**−0.30**	Enigmatic
paslaptingas	0.04	0.11	−0.02	0.26	−0.26	Mysterious
…	…	…	…	…	…	…

*Loadings with absolute values of 0.3 or greater are given in bold type*.

The component labeled Inducing Respect vs. a Sense of Danger in others (1/5, 8.2% of the total variance) has five subcomponents and describes the extent to which: (a) people appreciate us [e.g., appreciated (*brangus kitiems*) and arouses curiosity (*i̧domus*) vs. pathetic (*apgailėtinas*) and abominable (š*lykštus*)]; (b) we are able to motivate and encourage others to take up challenges [e.g., motivating (*motyvuojantis*), encouraging (*padra̧sinantis*) and educating (*lavinantis kitus*) vs. demotivating (*demotyvuojantis*), terrifying (*kraupus*), and deterrent (*atgrasus*)]; (c) we are able to influence others in a positive way and make them change their mind [e.g., persuasive (*sugebantis i̧kalbeti*) and influential (*i̧taigus*) vs. threatening (*grėsmingas*) and frightful (*klaikus*)]; (d) we are able to help others to control and direct emotions to their advantage [e.g., cooling somebody down (*atvėsinantis kitu̧ emocijas*), stabilizing (*stabilizuojantis*), and creating a sense of security (*saugus*)]; and (e) we gain the respect of others [e.g., respected (*gerbiamas*) and trustworthy (*patikimas*)].

The Attractiveness vs. Repulsiveness (2/5, 7.2% of the total variance) dimension includes three subcomponents, of which the first one describes to what extent we perceive ourselves to be sexually attractive to others [e.g., desirable (*geidžiamas*), alluring (ž*avus*), exciting (*jaudinantis*), and enticing (*gundantis*) vs. undesirable (*negeidžiamas*), unsexy (*neseksualus*), unattractive (*nepatrauklus*), and unattractive (*nesimpatiškas*)]. The second subcomponent mainly emphasizes the perceived cognitive reactions of other people to our personality—to what extent other people pay attention to us [distinct (*ryškus*), noticeable (*pastebimas*), recognizable (*atpaži̧stamas*), and memorable (*isimintinas*) vs. indistinct (*neryškus*), unnoticeable (*nepastebimas*), unrecognizable (*neatpaži̧stamas*), and unknown (*nežinomas*)]. Finally, the third facet reflects the extent to which our impact on others is captivating and enchanting [e.g., irresistible (*pavergiantis*), stunning (*stulbinantis*), intriguing (*intriguojantis*), provocative (*provokuojantis*), and impressive (*i̧spūdingas*)]. Thus, the second dimension from the five-component solution indicates that Attractiveness has cognitive, volitional, and motivational aspects.

The third component labeled Disintegrating vs. Integrating Impact on others (3/5, 5.4% of the total variance) defines the reactions that our personality evokes in the company of other people. The first subcomponent reflects the extent to which, in our mind, we are putting others in a good mood [e.g., entertaining (*pralinksminantis*), making somebody laugh (*sugebantis prajuokinti*), and funny (*juokingas*) vs. making somebody gloomy (*niūrinantis*), exhausting (*išsekinantis*), and make somebody angry (*užrūstinantis*)]. Quite a similar factor was observed by Saucier (2010) at the three-factor level of the social-effect structure with the highest loading in terms of entertaining, amusing, and hilarious. The second facet describes the extent to which, in our opinion, we have an ability to soothe the group [e.g., softening somebody (*sušvelninantis kitus*), supportive (*palaikantis*), and facilitating something for somebody (*palengvinantis kažka̧ kitiems*) vs. driving a wedge between somebody (*supriešinantis*), overtiring (*nukankinantis*), and hateful (*nekenčiamas*)]. Finally, the third subcomponent indicates the extent to which we are able to please others [e.g., worming oneself into somebody's favor (*sugebantis i̧sigerinti*), worming oneself into somebody's good graces (*sugebantis i̧siteikti*), and pleasing (*sugebantis i̧tikti*)].

The fourth component of the Soothing vs. Vexing effect (4/5, 5.2% of the total variance) includes three facets. The first subcomponent describes the extent to which we are able to cause harm to or enrich people [e.g., causing pain (*skaudinantis*), insulting (*i̧žeidžiantis*), and troublesome (*trikdantis*) vs. enriching (*praturtinantis kitus*), inspiring (*i̧kvepiantis*), and making somebody emotional (*sujausminantis*)]. The second facet reflects the extent to which our effect on others is relaxing, calming, or creating tension [e.g., calming (*nuraminantis*) and relaxing (*atpalaiduojantis*) vs. making somebody furious (*sugebantis sukelti i̧tūži̧*) and nerve-racking (*keliantis i̧tampa̧*)]. The third subcomponent, in turn, describes the perceived degree to which, we believe, we disturb others [e.g., disturbing (*trukdantis*), bothering (*kvaršintojas*), distracting somebody (*išblaškantis*), tiresome (*varginantis*), irritating (*erzinantis*), and annoying (*i̧kyrus*)].

The fifth component of Being Misunderstood by others (5/5, 3% of the total variance), which mainly describes the cognitive reactions of other people, consists of two facets not previously reflected by other dimensions. The first subcomponent describes the perceived degree of being an incomprehensible person, who causes some trouble to others [e.g., bizarre (*keistas*), unclear (*neaiškus*), and problematic (*keblus*)]. The second facet reflects the extent to which we are perceived as inscrutable [e.g., intricate (*sudėtingas*), complicated (*komplikuotas*), and enigmatic (*mi̧slingas*)].

To examine the proportion of the various social-effect types and their consistency with the content of the five main components, we explored in more detail the highest loading terms for each dimension. The emotional component was predominant in the first, third, and fourth components (50, 90, and 65% of the 20 highest loading terms, respectively), whereas terms referring to inducing respect from the first dimension mainly included reputational aspects and constituted 35% of the highest loading terms. In the second self-rating dimension, the proportions of cognitive, emotional, and motivational effects were comparable (45, 40, and 35% of the 20 highest loading terms, respectively), while the fifth dimension was mainly described by cognitive (50% of the 14 highest loading terms) and emotional (43% of the 14 highest loading terms) effects. Our findings regarding social-effect types were consistent with the content of the five social-effect dimensions.

At the 6, 9, and 11-component levels, which achieved the orthogonal-oblique best-match correlations, all the new dimensions had a too small number of the highest loading terms— <6—with an absolute loading of over 0.3. Based on the interpretability and saturation of the components, as well as the robustness indices, the five-component solution should be considered the most informative structure of social effects for the self-rating data set.

#### Social Effects as a Consequence of Personality Dispositions

To identify which personality dispositions of persons who provide self-ratings evoke social effects in other people, we calculated the linear correlation coefficients between the component scores of the one to five-component solutions and the personality dimensions measured by IPIP-BFM-50 and HEXACO-60 (refer to Table 7). Here, we will discuss in detail only the relations between the most informative five-component social-effect structure and the personality dimensions.

**Table 7:** Perceived social effects upon personality dispositions.

		**IPIP–BFM (*****N*** **= 96)**					**HEXACO (*****N*** **= 107)**					
**S/C**	**Lithuanian social-effect components**	**EXT**	**AGR**	**CON**	**NEU**	**INT**	**EXT**	**AGR**	**CON**	**EMO**	**OPN**	**HON**
1/1	General social-effect component	0.14	**0.64**	**0.35**	−0.09	**0.48**	**0.38**	0.27	0.21	0.24	**0.31**	0.07
1/2	Supportiveness vs. destructiveness	−0.04	**0.64**	**0.35**	0.01	**0.46**	0.16	**0.33**	0.22	0.18	**0.32**	0.22
2/2	Attractiveness vs. repulsiveness	**0.39**	0.09	0.06	−0.23	0.11	**0.51**	−0.02	0.06	0.19	0.08	−0.24
1/3	Supportiveness vs. destructiveness	−0.02	**0.61**	**0.35**	0.08	**0.46**	0.13	0.25	0.17	0.12	**0.36**	0.17
2/3	Attractiveness vs. repulsiveness	**0.39**	0.05	0.05	−0.14	0.11	**0.46**	−0.11	0.00	0.12	0.11	−0.29
3/3	Likability vs. being problematic	0.00	0.18	0.05	**−0.33**	0.04	0.25	**0.30**	0.22	0.26	−0.05	0.12
1/4	Eliciting sense of safety vs. sense of danger	−0.08	**0.58**	**0.32**	0.12	**0.38**	0.13	0.14	−0.03	0.14	0.17	0.05
2/4	Attractiveness vs. repulsiveness	**0.42**	0.05	0.06	−0.14	0.13	**0.45**	−0.09	0.07	0.09	0.19	−0.26
3/4	Soothing vs. vexing	0.09	**0.29**	0.15	−0.20	0.27	0.13	**0.37**	**0.43**	0.13	**0.33**	0.28
4/4	Being misunderstood	0.08	−0.04	0.02	0.23	0.12	−0.25	−0.12	0.05	−0.26	**0.30**	0.06
1/5	Inducing respect vs. sense of danger	−0.05	**0.45**	**0.38**	0.10	**0.37**	0.04	−0.04	0.07	0.13	0.09	0.09
2/5	Attractiveness vs. repulsiveness	**0.42**	0.03	0.04	−0.17	0.11	**0.47**	−0.07	0.06	0.11	0.16	−0.25
3/5	Disintegrating impact	0.03	**−0.44**	−0.07	−0.06	−0.22	−0.19	**−0.32**	0.08	−0.04	−0.23	0.02
4/5	Soothing vs. vexing	0.10	0.25	0.13	−0.22	0.23	0.13	**0.36**	**0.43**	0.13	**0.30**	0.27
5/5	Being misunderstood	0.13	−0.04	−0.03	0.20	0.10	−0.20	−0.06	0.05	−0.27	**0.36**	0.03

*Correlation coefficients with absolute value higher than 0.26 are statistically significant at p < 0.01 and are given in bold type. S/C, solution/component; EXT, Extraversion; AGR, Agreeableness; CON, Conscientiousness; NEU, Neuroticism; INT, Intellect; EMO, Emotionality; OPN, Openness; H-H, Honesty-Humility*.

The clearest correlations were found for only three social-effect dimensions. Hence, Attractiveness vs. Repulsiveness was related to Extraversion from both instruments, with correlation coefficients of 0.42 (*p* < 0.001) for IPIP-BFM-50, and 0.47 (*p* < 0.01) for HEXACO. Similarly, the component of the Disintegrating vs. Integrating Impact on others was most strongly related to the Big Five Agreeableness (*r* = −0.44, *p* < 0.001), as well as the HEXACO Agreeableness (*r* = −0.32, *p* < 0.001). Also, the social-effect dimension of Being Misunderstood by others showed the strongest correlation with the HEXACO Openness (*r* = 0.36, *p* < 0.001), and no statistically significant relations with any of the Big Five factors. Whereas, a social-effect dimension of Inducing Respect vs. a Sense of Danger was most strongly related to three Big Five factors: Agreeableness (*r* = 0.45, *p* < 0.001); Conscientiousness (*r* = 0.38, *p* < 0.001); and Intellect (*r* = 0.37, *p* < 0.001), in the case of HEXACO no statistically significant correlations between this social-effect component and personality dispositions were observed. Contrastingly, the social-effect component of the Soothing vs. Vexing effect had poor relations with the Big Five factors, but in the context of HEXACO showed correlations with Agreeableness (*r* = 0.36, *p* < 0.001), Conscientiousness (*r* = 0.43, *p* < 0.001), and Openness (*r* = 0.3, *p* < 0.001).

#### Lithuanian Self-Rating Social-Effect Dimensions Compared With the English and Croatian Social-Effect Lexicon Structures

To test the resemblance between the Lithuanian social-effect self-rating structure and the respective English and Croatian structures, we computed the linear coefficient of the correlations between the Lithuanian one to five-component scores and the marker scales of the English and Croatian social-effect dimensions (refer to Table 8).

**Table 8:** The relationship between Lithuanian, English, and Croatian lexicons of social effects in the self-rating data.

		**English SE lexicon**		**Croatian SE lexicon**		
**S/C**	**Lithuanian social-effect components**	**E1**	**E2**	**C1**	**C2**	**C3**
1/1	General social-effect component	**0.59**	**−0.79**	0.30	**−0.57**	**0.75**
1/2	Supportiveness v. destructiveness	0.27	**−0.82**	−0.10	**−0.57**	**0.57**
2/2	Attractiveness vs. repulsiveness	**0.75**	−0.14	**0.85**	−0.14	**0.53**
1/3	Supportiveness vs. destructiveness	0.21	**−0.74**	−0.11	−0.46	**0.52**
2/3	Attractiveness vs. repulsiveness	**0.66**	−0.03	**0.83**	−0.01	0.45
3/3	Likability vs. being problematic	0.42	−0.44	0.20	−0.47	0.38
1/4	Eliciting sense of safety vs. sense of danger	0.23	**−0.63**	−0.08	−0.49	**0.57**
2/4	Attractiveness vs. repulsiveness	**0.62**	−0.04	**0.82**	0.04	0.40
3/4	Soothing vs. vexing	0.20	**−0.56**	0.01	−0.30	0.20
4/4	Being misunderstood	−0.42	0.19	−0.26	0.42	−0.39
1/5	Inducing respect vs. sense of danger	0.16	**−0.51**	−0.09	−0.39	0.45
2/5	Attractiveness vs. repulsiveness	**0.65**	−0.03	**0.84**	0.03	0.40
3/5	Disintegrating impact	−0.21	0.41	−0.02	0.29	−0.36
4/5	Soothing vs. vexing	0.19	**−0.52**	0.02	−0.28	0.17
5/5	Being misunderstood	−0.37	0.20	−0.18	0.45	−0.39

*N = 203. Correlation coefficients with absolute values higher than 0.19 are statistically significant at p < 0.01. Coefficients with absolute values of 0.5 or greater are given in bold type. S/C, solution/component; SE lexicon, social-effect lexicon; E1, the extent to which a person is a source of pleasure to others; E2, the extent to which a person is a source of pain to others; C1, Attractiveness-Popularity; C2, Mysteriousness vs. irritation; C3, Likeability*.

As shown in Table 8, the Lithuanian and English two-component self-rating social-effect structures are highly convergent. The first English dimension reflecting the extent to which a person is a source of pleasure to others has a correlation of *r* = 0.75 with the Lithuanian Attractiveness vs. Repulsiveness. The second English dimension describing the extent to which a person is a source of pain to others shows a correlation of *r* = −0.82 with the Lithuanian Supportiveness vs. Destructiveness component.

The relations between the Lithuanian three-component self-rating social-effect structure and the respective Croatian structure (refer to Table 8) were not as clear as in the case of the English social-effect dimensions. We observed the only one-to-one correspondence between the Lithuanian Attractiveness vs. Repulsiveness and the Croatian Attractiveness-Popularity (*r* = 0.83), whereas the Lithuanian component of Supportiveness vs. Destructiveness was related to both the Croatian Likeability (*r* = 0.52) and Mysteriousness vs. Irritation (*r* = −0.46). Finally, the Lithuanian Likability vs. Being Problematic to others showed the highest relations with the Croatian Mysteriousness vs. Irritation (*r* = −0.47) and Likeability (*r* = 0.38), however, these correlations were slightly weaker.

## Discussion

In the present study, we aimed to explore the untapped potential of the psycholexical approach by focusing on the non-dispositional personality-relevant category of social effects and by including various word classes, namely the adjectives, type-nouns, attribute-nouns, participles, and verbs that are capable of describing human qualities. In this study, we defined social effects as psychological reactions, focusing on a wide spectrum of emotional, cognitive, and motivational states experienced by the observer upon the expression of personality qualities by an observed person.

Compared to previous studies on social effects, our research had several distinctive features. First of all, since we went beyond a single linguistic category, we used a more comprehensive pool of 208 social-effect descriptors for our analysis, whereas, in other research, scholars collected ratings on shorter lists—the 138 Croatian (Mlačić, 2016) and 32 English (Saucier, 2010) social-effect adjectives. Second, unlike in previous studies on social-effect descriptors, we precisely described a target in the observer-rating sample by controlling the attitude and the gender of the observed person. Finally, we controlled the gender of the respondents in each sample.

Even though in previous English (Saucier, 2010) and Croatian (Mlačić, 2016) studies the main social-effect factors were replicated across self- and observer-ratings, the current research showed that the perception of social effects may differ depending on the judgment perspective. Although solutions with one and two components were highly congruent across the two data sets, in the more fine-grained structures the dimensions that described the reactions of respondents to the personality dispositions of others diverged from the components referring to how other people react to the personality of the respondents. Thus, in the observer-rating data set, the most informative structure was a four-component solution with dimensions of (1) Supportiveness vs. Destructiveness, (2) Eliciting Fear, (3) Attracting Attention, and (4) Erotic Attractiveness. Whereas, in the self-rating data set the optimal structure included five dimensions of (1) Inducing Respect vs. a Sense of Danger, (2) Attractiveness vs. Repulsiveness, (3) Disintegrating Impact, (4) Soothing vs. Vexing effect, and (5) Being Misunderstood by others. Nevertheless, the observer-rating components explained a higher proportion of the variance compared to the self-rating dimensions.

Content analysis of the most informative observer- and self-rating social-effect structures show that while describing themselves, people perceive Attractiveness as a mixture of three aspects: attracting attention, captivation, and sexual desire, while from the observer-rating perspective, Attracting Attention and Erotic Attractiveness split into two separate dimensions. Some previous evidence shows that people, regardless of gender, tend to rate their partners as being more physically attractive than themselves (Swami et al., 2007), so Erotic Attractiveness might be more distinctive when we rate others compared to ourselves. Also, the component of Inducing a Sense of Danger contains additional content referring to Respect in the self-rating data set. If we define respect as a person's subjective assessment of how other people that share group membership evaluate them (Huo and Binning, 2008), and recognition of respect usually comes from the appraisal of personality-related attributes (Darwall, 1977), then the occurrence of respect-related terms is more natural in the self-rating sample where people evaluate how others react to their personality. Inducing a Sense of Danger on the opposite pole of Inducing Respect might be caused by the fact that in some age groups, disrespect enhances the risk of revengeful violence used to regain lost social status (Anderson, 1994). One more difference between the self- and observer-rating dimensions is that the self-rating component of Disintegrating vs. Integrating Impact refers to the effect on the group, whereas the observer-rating dimension of Supportiveness vs. Destructiveness, which at the first glance seems to express quite a similar meaning, has more individual character. Again, this difference might be explained by the specificity of the self- and observer-rating perspectives. Thus, observer-raters describe their own reactions to the personality dispositions of a familiar person, so in general, they express an individual effect. While self-raters evaluate how other people react to their personalities, respondents from this sample might keep in mind that their personality characteristics affect a whole group.

A possible reason for the divergent self- and observer-rating structures could be the fact that each of the two perspectives involves different cognitive processes. Providing self-ratings on social effects is a somewhat harder task as the respondents need to express their opinions on the psychological reactions experienced by others upon their personality dispositions. This might require the involvement of self-schemata—“cognitive generalizations about the self, derived from past experience, that organize and guide the processing of self-related information contained in the individual's social experiences” (Markus, 1977, p. 64). Additionally, respondents have to resist succumbing to self-enhancement bias which is not so common in the case of observer-ratings (refer to Krueger, 1998). In turn, the description of social effects on the personality of a familiar observed person requires expressing psychological states that were experienced at some point by the respondent. This mostly involves objective self-awareness (Duval and Wicklund, 1972) which helps in recognizing and understanding our own emotional and cognitive states, as well as motives and desires. Overall, there is a fundamental asymmetry in the knowledge of self and others (Moran, 2001) which might produce different self- and observer-rating social-effect structures.

Compared with the results from previous studies, the present research provides evidence for the replication of the English social-effect dimensions (Saucier, 2010) in the Lithuanian two-component solution in both data sets, with a slight re-alignment of the Lithuanian dimensions in the observer-rating perspective. Thus, the Lithuanian two-component structure, similarly to the English dimensions, reflects the hedonic principles of maximizing pleasure and avoiding distress (refer to Young, 1967) with one dimension describing the extent to which a person is a source of pleasure to others, and the other component expressing the extent to which a person is a source of distress to others. However, at the more detailed levels of the Lithuanian structure, some fine-grained components are not strongly related to the two main English social-effect dimensions, such as the observer-rating component of Attracting Attention or the self-rating dimension of Being Misunderstood by others. Analysis of the 32 English social-effect terms showed that most of the highest loading descriptors of these two Lithuanian components were not included in the English social-effect set. The possible reason could be the weaker emphasis on cognitive effects in the English study, whereas, in the Lithuanian structure, cognitive reactions mainly constitute the components of Attracting Attention and Being Misunderstood by others. Also, the Lithuanian study resulted in more elaborate social-effect structures compared to the English research. Overall, the weaker internal replication of the English social-effect factors in solutions with more than three dimensions could generally be caused by the small number of social-effect descriptors that were included in the analysis (refer to Barelds and De Raad, 2015).

Further comparisons showed that three main Croatian social-effect factors (Mlačić, 2016) were not fully recovered in the Lithuanian three-component solution regardless of the description perspective. The only replicated Croatian dimension was Attractiveness-Popularity which had its equivalent in the component of Attractiveness vs. Repulsiveness, both dimensions at least partly referring to cognitive effects. The two remaining Croatian factors were related to the same Lithuanian component of Destructiveness vs. Supportiveness, whereas the Lithuanian dimensions of Activating vs. Oppressing Impact (observer-rating data set) and Likability vs. Being Problematic to others (self-rating data set) did not have their clear equivalents in the Croatian social-effect structure. At the more fine-grained Lithuanian observer-rating levels, there was no one-to-one correspondence between the Croatian and Lithuanian factors, whereas, in the self-rating data set, Attractiveness-Popularity was recovered in the Lithuanian component of Attractiveness vs. Repulsiveness. Also, the Croatian dimension labeled Likeability was replicated in the Lithuanian component of Eliciting a Sense of Safety vs. a sense of Danger in others at the four-dimension self-rating level. Accordingly, as with the English study, the Croatian research resulted in a structure with broader factors, probably because of the smaller number of selected variables (138) compared to the current study (208). Also, the Croatian dimensions could be represented diffusely in the respective Lithuanian structure because of the different conceptualization of social effects. As discussed previously, the proportion of types of social reactions was different in the Croatian and Lithuanian languages, which could impact the resulting social-effect structures.

The content analysis of the Lithuanian two-component social-effect solutions in both perspectives confirms previous findings in the English study (Saucier, 2010). Thus, two broad emic social-effect components resemble the personality structure of the Big Two, with one component emphasizing some aspects of Dynamism, and the other dimension reflecting Social Self-Regulation (refer to Saucier et al., 2014; De Raad et al., 2018a). Also, the correlations found between the social-effect dimensions and the personality structures with five and six factors to some extent confirm the transactional approach to personality dispositions (Saucier, 2010) assuming that stable human qualities emerge from the transaction between a person and their social environment. Hence, the dimensions reflecting Attractiveness vs. Repulsiveness, Attracting Attention, or Activating vs. Oppressing Impact in most emic solutions have the strongest relations with IPIP-BFM-50 and HEXACO-60 Extraversion regardless of the perspective. Since IPIP-BFM-50 Extraversion mainly describes Talkativeness and Social Self-Esteem (Goldberg, 1992), and HEXACO-60 Extraversion reflects Social Self-Esteem, Social Boldness, Sociability, and Liveliness (Ashton and Lee, 2009), the behavioral pattern of a highly extraverted person will naturally attract others' attention, as well as activate people from their social environment to interact with each other. Other relations between social-effect components and personality dimensions differ depending on the description perspective.

In the observer-rating data set, the dimension of Supportiveness vs. Destructiveness shows the highest correlations with IPIP-BFM-50 Agreeableness and Intellect and HEXACO-60 Agreeableness and Honesty at different levels of the social-effect hierarchy. These results are in line with the content of the personality dimensions mentioned above. Thus, the expression of Intellect might provide instructions and suggestions for improvement in different areas, whereas the behavioral patterns of a highly agreeable and honest person, who is generally helpful, peaceful, forgiving, gentle, patient, sincere, and fair (refer to De Raad and Peabody, 2005; Ashton and Lee, 2009), would uplift other people socially and morally. Also, the observer-rating component of Eliciting Fear negatively correlates with IPIP-BFM-50 Agreeableness which means that the behavioral patterns of a person who does not care about the feelings and problems of others and often insults people (Goldberg, 1992) could evoke a sense of danger in the social environment. However, the correlation is rather weak. Interestingly, the component of Erotic Attractiveness does not show any strong correlations with personality dimensions, which contradicts the idea that physically attractive people can be perceived as possessing positive personality dispositions (Barocas and Karoly, 1972).

Overall, in the self-rating data set, the social-effect dimensions are represented more diffusely in the five-factor and six-factor personality structures compared to the observer-rating data set. Also, most of the correlations between social-effect self-rating components and personality dimensions are weaker than the analogous relations in the observer-rating data set. This might corroborate our assumption that the observer-rating perspective is more natural for describing social effects, and in the case of self-descriptions, respondents have a harder task that demands the involvement of more complex cognitive processes. Nevertheless, some interesting relations are worth noting. First of all, the component of Likability vs. Being Problematic to others has the strongest relation with IPIP-BFM-50 Neuroticism and HEXACO-60 Agreeableness. Since Agreeableness from the HEXACO model has, in general, some features of the Neuroticism of the Big Five, e.g., losing temper quickly or irritability (refer to Ashton et al., 2004; Saucier, 2009), people who believe they are emotionally stable evoke likability in their social environment, whereas those who attribute high emotional volatility to themselves may experience that they cause problems to others. Also, people with lower results on IPIP-BFM-50 and HEXACO-60 Agreeableness also believe that they have a disintegrating impact on others, which is in line with the content of the Agreeableness factor. Finally, persons with higher results for Openness, feel misunderstood by others. In general, people who score high for Openness have inquisitive minds, use their imagination, have unusual and unconventional ideas (refer to Ashton and Lee, 2009) which might not be accepted by most people, so these results stay in line with the idea that personality is “an individual's footprint on the social world” (Saucier, 2010, p. 224).

Including different word classes in the present study shows that although almost “invisible” in the psycholexical approach, verbs, and participles that are derived from verbs can play a crucial role in describing individual differences beyond personality dispositions. Since social effects are more transitional by their nature compared to relatively stable dispositions, they need adequate resources in terms of parts of speech. Thus, verbs that have a less durable character compared to adjectives and nouns, and to some extent get their meaning from interpersonal interactions (refer to De Raad, 2000), can be a better choice for expressing some aspects of social effects. In the current study, in two of the four observer-rating components, the largest group describing social effects is participles that are derived from verbs, and in the two remaining dimensions, the number of adjectives and participles is almost equal. Whereas, in the most informative self-rating solution, participles prevail in four of the five dimensions, and only the component of Being Misunderstood by others has the largest group of adjectives. Interestingly, type-nouns play almost no role in describing social effects. Our findings corroborate the idea that narrowing the research to a one-word class of adjectives makes the psycholexical approach suboptimal as some aspects of the description of individual differences might be missed. Also, a longer list of terms results in more detailed lexical structures (refer to Barelds and De Raad, 2015), so there is a need to include different word classes to get more fine-grained lexical factors.

A possible limitation of this study is that only two judges participated in the selection of personality-relevant verbs. Although both assessors have extensive experience in the taxonomy of East Slavic (Ukrainian, Belarusian, and Russian) and West Slavic (Polish) languages, the small number of judges could have partially affected the representativeness of the set of social-effect verbs. Another limitation is the fact that we used student samples. Although it is common to recruit students in psycholexical research, generalizing our results to the Lithuanian population could be limited. In the future, an additional study with a more representative sample could verify the findings of the current study. Also, recruiting larger samples could help to examine the differences in the social-effect solutions depending on the age and gender of the participants. Finally, due to space limitations, we did not present structures that derived from the original data set. Future research could focus on analyzing so far unexplored social-effect lexicons in terms of different word classes and comparing social-effect factors in various natural languages.

## Conclusion

Overall, our findings corroborate the transactional approach to personality and the idea that psycholexical research needs to be extended beyond dispositional adjectives. Thus, individual differences should not be narrowed down to differences within personality dispositions. From the perspective of psychology, to better predict our behavior toward other people, it is important to take into account the social effects that also distinguish people from one another. The current study also shows that self-observations might differ from the descriptions of others, and the observer-ratings could play a dominant role in some domains of individual differences. Finally, based on the different inter-judge consistency for the subcategory of social effects among various studies, as well as the different proportions of social-effect types in the examined natural languages, we assume that the definition of social effects needs to be refined.

## Data Availability Statement

The datasets presented in this article are not readily available because the psycholexacal project funded by the NCN is not yet completed. Requests to access the datasets should be directed to ana.ivanova.kul@gmail.com.

## Ethics Statement

Ethical review and approval was not required for the study on human participants in accordance with the local legislation and institutional requirements. Written informed consent for participation was not required for this study in accordance with the national legislation and the institutional requirements.

## Author Contributions

AV collected data and organized the database. AV and OG performed the statistical analysis. AV wrote the first draft of the manuscript. OG and BM wrote sections of the manuscript. All authors contributed to conception, design of the study, contributed to manuscript revision, read, and approved the submitted version.

## Funding

We gratefully acknowledge funding from the National Science Centre (Poland) (Grant No. UMO-2017/25/N/HS6/02210) to AV. The funder had no role in study design, data collection and analysis, decision to publish, or preparation of the manuscript.

## Conflict of Interest

The authors declare that the research was conducted in the absence of any commercial or financial relationships that could be construed as a potential conflict of interest.

## Publisher's Note

All claims expressed in this article are solely those of the authors and do not necessarily represent those of their affiliated organizations, or those of the publisher, the editors and the reviewers. Any product that may be evaluated in this article, or claim that may be made by its manufacturer, is not guaranteed or endorsed by the publisher.

## Footnotes

^1^The sum of the percentages exceeds 100% because some of the terms describe several social-effect types at the same time.

^2^The original Lithuanian terms and loadings are given in the round brackets.
