# Supplementary material for: The Impact of Our Personality on Others: The Lithuanian Comprehensive Lexical Taxonomy of Social Effects
Source: Front Psychol. 2022 Apr 25;13:869920. doi: 10.3389/fpsyg.2022.869920 (PMC9083117; doi:10.3389/fpsyg.2022.869920)
Supplement: Supplementary file 1 [file Data_Sheet_1.docx]

Appendix 1

Re-rotated two-component structure in the observer-rating data

| Destructiveness vs Supportiveness (1/2`):   - setting others at variance (*sugebantis_„supjudyti”*, 0.60), a wedge putting between sb (*supriešinantis*, 0.60), irritating (*dirginantis*, 0.59), nerve-racking (*keliantis_įtampą*, 0.59), harmful (*kenkiantis*, 0.58), abasing sb (*žeminantis_kitus*, 0.56), causing pain (*skaudinantis*, 0.55), a wedge between sb (*sugebantis_sukiršinti putting*, 0.55), making sb furious (*sugebantis_sukelti_įtūžį*, 0.55), make sb angry (*užrūstinantis*, 0.54)   versus   - encouraging sb (*padrąsinantis*, -0.68), calming (*nuraminantis*, -0.68), supportive (*palaikantis*, -0.68), trustworthy (*patikimas*, -0.68), coherent (*suprantamas*, -0.67), not tiring (*nevarginantis*, -0.63), safe (*saugus*, -0.62), elating (*sukeliantis_kitiems_laimę*, -0.60), harmless (*nekenksmingas*, -0.60), not causing stress to sb (*nesukeliantis_įtampos_kitiems*, -0.59), |
| --- |
| Attractiveness vs Repulsiveness (2/2`):   - impressive (*įspūdingas*, 0.70), appealing (*žavus*, 0.66), attractive (*patrauklus*, 0.64), charming (kerintis, 0.64), attractive (*pritraukiantis*, 0.61), desirable (*geidžiamas*, 0.61), enticing (*gundantis*, 0.61), alluring (*viliojantis*, 0.60), likeable (*simpatiškas*, 0.60), stunning (*pritrenkiantis*, 0.58), exciting (*jaudinantis*, 0.57),   versus   - unattractive (*nepatrauklus*, -0.71), unlikeable (*nesimpatiškas*, -0.67), repulsive (*neviliojantis*, -0.67), undesirable (*negeidžiamas*, -0.66), not sexy (*neseksualus*, -0.66), untinteresting (*neįdomus*, -0.66), indistinct (*neryškus*, -0.64), boring (*nuobodus*, -0.64), unlovable (*nemielas*, -0.63), pestering sb (*įgrystantis*, -0.60), |

*Notes*. The appendix presents descriptors with the highest positive and negative factor loadings.

Appendix 2

The structure of the social-effect descriptors in relation to the type of psychological aspect

| Language | | Dimensions | Emo, % | Cog, % | Mot, % | Rep, % | Oth, % |
| --- | --- | --- | --- | --- | --- | --- | --- |
| LT SE lexicon | self-ratings | Inducing Respect vs Sense of Danger | 50.00 | 5.00 | 15.00 | 35.00 | 0 |
|  |  | Attractiveness vs Repulsiveness | 40.00 | 45.00 | 35.00 | 0 | 10.00 |
|  |  | Disintegrating Impact | 90.00 | 0 | 5.00 | 5.00 | 0 |
|  |  | Soothing vs Vexing | 65.00 | 10.00 | 0 | 25.00 | 0 |
|  |  | Being Misunderstood | 42.86 | 50.00 | 0 | 7.14 | 7.14 |
|  | peer-ratings | Supportiveness vs Destructiveness | 65.00 | 10.00 | 10.00 | 15.00 | 5.00 |
|  |  | Eliciting Fear | 85.00 | 0 | 5.00 | 15.00 | 0 |
|  |  | Attracting Attention | 15.00 | 80.00 | 15.00 | 0 | 5.00 |
|  |  | Erotic Attractiveness | 40.00 | 25.00 | 40.00 | 0 | 15.00 |
| EN SE lexicon |  | Being source of pleasure | 52.38 | 23.81 | 9.52 | 14.29 | 4.76 |
|  |  | Being source of pain | 81.82 | 9.09 | 0 | 9.09 | 0 |
| HR SE lexicon |  | Attractiveness-Popularity | 31.03 | 41.38 | 27.59 | 17.24 | 6.90 |
|  |  | Mysteriousness-Irritation | 33.33 | 29.63 | 0 | 14.81 | 29.63 |
|  |  | Likeability | 50.00 | 0 | 0 | 28.57 | 21.43 |

*Notes*. LT SE lexicon – Lithuanian social-effect lexicon, EN SE lexicon – English social-effect lexicon, HR SE lexicon – Croatian social-effect lexicon, Emo – emotional reactions, Cog – cognitive reactions, Mot – motivational reactions, Rep – reputational aspects of social effects, Oth – other aspects of social reactions. The sum of the percentages per component might exceed 100% because some of the terms describe several social-effect types at the same time.
